# Comparative Efficacy and Safety of Different Low‐Dose Platelet Inhibitors in Patients With Coronary Heart Disease: A Bayesian Network Meta‐Analysis

**DOI:** 10.1111/jebm.12671

**Published:** 2024-12-21

**Authors:** Chunxing Li, Zhao Ren, Jia Liu, Shuo Liang, Hua Liu, Dongxiao Wang, Yue Wang, Yumin Wang

**Affiliations:** ^1^ Department of Pharmacy Aerospace Center Hospital, Peking University Aerospace School of Clinical Medicine Beijing China; ^2^ Zi Zhu Yuan Community Healthcare Center Aerospace Center Hospital Beijing China; ^3^ Department of Respiratory and Critical Care Medicine Aerospace Center Hospital, Peking University Aerospace School of Clinical Medicine Beijing China

**Keywords:** bleeding, coronary heart disease, low‐dose, myocardial infarction, network meta‐analysis, platelet aggregation inhibitors

## Abstract

**Objective:**

The optimal low‐dose antiplatelet agents in patients with coronary heart disease (CHD) had not been determined. The objective of this study was to compare the impact of different low‐dose antiplatelet agents on cardiovascular outcomes and bleeding risks in patients with CHD.

**Methods:**

We searched PubMed, Embase, the Cochrane Library, China National Knowledge Infrastructure, VIP, WanFang Data, and China Biology Medicine. Randomized controlled trials (RCTs) enrolling patients with CHD treated with different low‐dose platelet aggregation inhibitors were included. The revised Cochrane Risk of Bias Tool for Randomized Trials Risk was used to assess risk of bias in RCTs. A Bayesian random network meta‐analysis (NMA) was conducted, with odds ratios (OR) and 95% confidence intervals (CI) as effect estimates in R 4.2.2 software and Stata 15.0. The quality of evidence was assessed using the Confidence in NMA framework.

**Results:**

Sixteen RCTs involving 6350 patients were included. All participants were treated with a recommended dose of aspirin plus a low or standard dose of P2Y12 receptor antagonist. Low‐level evidence indicated the risk of major adverse cardiovascular events (MACE) was similar among low doses of prasugrel, ticagrelor, standard doses of prasugrel, ticagrelor, and clopidogrel. Low‐ to moderate‐level evidence suggested there was no difference in bleeding risk among low dose of prasugrel, ticagrelor, clopidogrel compared to standard dose of prasugrel, ticagrelor, and clopidogrel. NMA showed that low dose of prasugrel had the highest probability of being the best intervention in terms of MACE, myocardial infarction, and bleeding events leading to discontinuation.

**Conclusion:**

Based on low‐level evidence, low dose of prasugrel combined with standard dose of aspirin can be recommended for patients with CHD, low dose of ticagrelor was similar in terms of MACE and bleeding compared with standard dose of P2Y12 receptor antagonist.

The systematic review was registered in PROSPERO with the registration number CRD42023438376.

## 1 Introduction

Coronary heart disease (CHD) is a widespread cardiovascular ailment marked by the buildup of atherosclerotic plaques within the coronary arteries. As living standards have improved, CHD has evolved into a global health crisis and a primary contributor to illness and premature mortality on a worldwide scale [1]. Antiplatelet agents, by inhibiting platelet aggregation, effectively hinder the development of intracoronary thrombi and reduce the likelihood of adverse cardiovascular incidents, playing a pivotal role in CHD treatment [2]. The guidelines established by the American College of Cardiology [3] and the European Society of Cardiology [4] vigorously advocate the use of antiplatelet agents in managing CHD. However, while antiplatelet agents offer substantial cardiovascular advantages, safety concerns, particularly regarding bleeding events such as fatal or major bleeding, remain significant.

Due to concerns about bleeding, clinicians and patients often tend to use lower doses of antiplatelet drugs as a potential strategy for mitigating bleeding risks. Studies have indicated that lower doses of antiplatelet drugs demonstrate comparable efficacy to standard doses, with a similar or slightly increased risk of bleeding [5, 6, 7]. Currently, common antiplatelet drugs used in clinical practice include aspirin, clopidogrel, prasugrel, ticagrelor, cilostazol, dipyridamole, and indobufen. Studies have shown that different antiplatelets may have different bleeding risks. Clopidogrel monotherapy, compared with aspirin monotherapy, was associated with lower rates of the composite net clinical outcome in patients without clinical events for 12±6 months after percutaneous coronary intervention (PCI) with drug‐eluting stents [8]. In patients requiring indefinite antiplatelet monotherapy after PCI, clopidogrel monotherapy was superior to aspirin monotherapy [9]. Clopidogrel monotherapy significantly reduced the risk of the composite of all‐cause death (ACD), nonfatal myocardial infarction (MI), stroke, readmission due to acute coronary syndrome (ACS), and Bleeding Academic Research Consortium (BARC) bleeding type 3 or greater [9]. The Global Leaders trial showed that the ischemic composite endpoint was reduced by 26% with ticagrelor monotherapy compared with aspirin monotherapy. In contrast, type 3 or 5 bleeding, as defined by BARC, was increased with ticagrelor monotherapy [10]. Patients who received a P2Y12 inhibitor had a borderline reduction in the risk of MI compared with those who received aspirin. The risks of stroke, ACD, and vascular death were comparable. Similarly, the risk of major bleeding did not differ between patients who received P2Y12 inhibitor and those who received aspirin [11]. Compared with the clopidogrel cohort, the newer P2Y12 cohort had lower rates of early mortality and a similar risk of major bleeding [12].

However, most published studies typically compared the efficacy and safety of two antiplatelet agents, often neglecting the simultaneous comparison of three or more agents [5, 6, 7]. Only one RCT had compared the efficacy and safety of two different low‐dose antiplatelet regimens [13]. Consequently, among numerous antiplatelet treatment options, identifying the drug with the lowest risk of bleeding and the one with the most superior efficacy remained a matter of important concern for both clinicians and patients. NMA was an increasingly popular tool for simultaneously comparing the efficacy or safety of three or more interventions [14]. Therefore, we performed a network meta‐analysis (NMA) to evaluate the efficacy and safety of different low‐dose antiplatelet drugs, intending to identify the optimal antiplatelet drug therapy.

## 2 Methods

We performed an NMA according to a prospectively registered protocol (PROSPERO No. CRD42023438376), and the results were reported following the Preferred Reporting Items for Systematic Reviews and Meta‐Analyses extension statement for NMA [15].

### 2.1 Data Sources

We searched PubMed, Embase, and the Cochrane Central Register of Controlled Trials, China National Knowledge Infrastructure, VIP, WanFang Data, and China Biology Medicine using the search strategy developed with the help of a medical information specialist (Supplementary Material 1). ClinicalTrials.gov was also searched for unpublished or ongoing trials. Reference lists of included studies and relevant systematic reviews were screened for more studies. Searches were conducted from inception until December 27, 2022, and updated on January 29, 2024. Screening was conducted by two independent blinded reviewers (LCX and RZ) using Endnote X9 software. Disagreements were resolved by a third reviewer (WYM).

### 2.2 Eligibility Criteria

(1) Participants: participants with CHD, such as stable angina and ACS, unstable angina, non‐ST‐segment elevation myocardial infarction (NSTEMI), and ST‐segment elevation myocardial infarction (STEMI). (2) Interventions and controls: the intervention measures for the experimental group and the control group could be either low‐dose antiplatelet drugs (aspirin, indobufen, clopidogrel, prasugrel, ticagrelor, cilostazol, and dipyridamole) or standard‐dose antiplatelet drugs. The interventions were categorized as low or standard doses according to a priori‐defined cut‐off that was based on a summary of product characteristics and clinical trial dosing regimens (Table S2.1). (3) Outcomes: the primary efficacy outcome was major adverse cardiovascular events (MACE), which was defined as a composite of cardiovascular death (CVD), nonfatal MI, nonfatal stroke, stent thrombosis, or ischemia‐driven revascularization [16]. The primary safety outcome was bleeding, defined as all bleeding events. Additional outcomes included MI, ischemic stroke, CVD, ACD, major bleeding (major bleeding based on thrombolysis in myocardial infarction (TIMI) criteria [17] or type 3b based on BARC criteria [18]), minor bleeding (minor bleeding based on TIMI criteria or type 3a based on BARC criteria) [17, 18], minimal bleeding (minimal bleeding based on TIMI criteria or type 2 based on BARC criteria) [17, 18], and bleeding events leading to discontinuation or nonadherence. (4) Study design: randomized controlled trials (RCTs). The follow‐up time in terms of safety and efficacy outcomes was at least 1 and 6 months, respectively.

### 2.3 Data Extraction

We extracted data with a standardized form that was previously tested in a pilot study. Data included study characteristics (country, length of follow‐up, number of patients, events reported) and patient characteristics (mean or median age in years, percentage of females, baseline body mass index (BMI) or weight, comorbidities and concomitant medication). Data on the interventions included low‐dose platelet aggregation inhibitors. Two reviewers (LCX and RZ) independently completed all data extractions, which were then verified by a third reviewer (WYM).

### 2.4 Quality Assessment

Two investigators (LCX and RZ) independently assessed the quality of the included studies according to the revised Cochrane Risk of Bias Tool for Randomized Trials (RoB version 2.0) [19]. This method considered the study's randomization process, bias due to deviations from intended interventions, missing outcome data, measurement of the outcome, and selection of the reported result. Each domain was classified qualitatively as having a high, some concern, or low RoB. Finally, concerning “overall RoB,” studies were evaluated at low RoB in case all domains were deemed at low risk of potential biases, studies were evaluated at high RoB in case they had at least one domain deemed at high risk of potential biases.

Two independent investigators (LCX and RZ) evaluated the evidence's certainty using the CINeMA framework for NMA, with any disagreements being resolved by a panel of three researchers (LCX, RZ, and WYM). This framework was broadly based on the Grading of Recommendations, Assessment, Development and Evaluation (GRADE) framework and considers six domains: within‐study bias, reporting bias, indirectness, imprecision, heterogeneity, and incoherence. Each of these domains can be judged at three levels (no concerns, some concerns, or major concerns). Finally, judgments in the domains were summarized in a single confidence rating of “high,” “moderate,” “low,” or “very low.”

### 2.5 Statistical Analysis

For all outcomes, we summarized the efficacy and safety of interventions using OR and corresponding 95% CI. This NMA was based on a Bayesian approach using the Markov Chain Monte Carlo simulation with a thinning interval of 10. A random‐effects NMA was performed using the “rjags,” “gemtc,” and “ggplot2” packages with specific parameters: 50,000 simulation iterations and 10,000 adaptation iterations. Model selection and goodness‐of‐fit were evaluated through deviance information criteria. The trace plot, density plot, and Brooks–Gelman–Rubin diagnostic were performed to assess the convergence through visual inspection, with a potential scale reduction factor value of <1.05 considered an indication that the simulation was valid. To assess heterogeneity, we calculated *I* ^2^ statistics. The *I* ^2^ estimates and their 95% CI were used to assess heterogeneity: low (<25%), moderate (25%–50%), and high (>50%) [20]. The contribution matrix plot was used to display the contribution of each direct comparison to the NMA results within each comparison group.

We estimated the relative treatment rankings for each intervention according to the surface under the cumulative ranking curve (SUCRA) values, SUCRA plot, and score are presented in the results for all outcomes.

Network diagrams, contribution plots, and comparison‐adjusted funnel plots were performed using STATA (version 15.0; StataCorp, College Station, TX, USA). Trace plot, density plot, forest plot, and SUCRA plot were performed in R 4.2.2.

## 3 Results

### 3.1 Search Results

A total of 5525 articles were retrieved. After removing 944 duplicates, 4581 records were screened by reviewing titles and abstracts, resulting in the exclusion of 4134 records. Subsequently, 447 full‐text articles were assessed. Sixteen RCTs [13, 21, 22, 23, 24, 25, 26, 27, 28, 29, 30, 31, 32, 33, 34, 35], involving 6350 participants, met our inclusion criteria (Figure S3.1). The details of the included studies could be found in Supplementary Material 4.

### 3.2 Characteristics of Included Studies

Tables S5.1 and S5.2 displayed the characteristics of the included studies, with 14 (87.50%) studies conducted in Asia. The median number of patients was 61 (31–1170, interquartile range (IQR) = 83), and the mean age was 63.97 ± 6.27 years. The mean proportion of females was 27.67% ± 26.37%. The patients had primarily normal body weight, with a mean baseline BMI of 25.14±1.56 kg/m^2^.

Of the participants, 83.54% had received PCI. Within the studies included, seven (58.3%) focused on patients with ACS, four included both ACS and chronic coronary syndrome (CCS) patients, and two focused solely on CCS. The studies displayed significant variability in the percentages of patients with various forms of CHD, ranging from 14.6% to 67.5% for STEMI patients, 7.7% to 100% for NSTEMI patients, and 7.5% to 62.7% for unstable angina patients. The primary comorbid conditions were hypertension, diabetes, and dyslipidemia, with other comorbidities including chronic kidney disease, hepatic function disorder, and stroke. Ten studies reported on the use of concomitant medications, with seven indicating the use of proton pump inhibitors. The most common concomitant medications were beta‐blockers, angiotensin‐converting enzyme inhibitors or angiotensin receptor blockers, and statins. Other medications included calcium channel blockers.

All participants were treated with dual antiplatelet therapy (DAPT): a combination of standard‐dose aspirin plus a standard‐dose or low‐dose P2Y12 receptor antagonist. Nine studies were treated with low‐dose prasugrel (5 mg in four studies, 3.37 mg in four studies, and 7.5 mg in one study), and seven studies were treated with low‐dose ticagrelor (45 mg twice daily in four studies, 60 mg twice daily in three studies). One study was treated with low‐dose clopidogrel (50 mg), and four, five, and eight studies were treated with standard doses of prasugrel, ticagrelor, and clopidogrel, respectively. The median length of the intervention period in the studies was 48 weeks (range 4–144 weeks.).

### 3.3 Assessment of RoB

The quality of the studies varied (Supplementary Material 6). No studies had high RoB for deviations from the intended intervention, the measurement of the outcome, or the selection of the reported result. Two (12.50%) studies had a high RoB for the randomization process, and one (6.25%) had a high risk for missing outcome data. Overall, four studies (25.00%) had a low RoB, nine studies (56.25%) raised some concerns, and three studies (18.75%) had a high RoB (Supplementary Material 6.1). The RoB for each outcome was shown in the RoB chart (Supplementary Material 6.2).

### 3.4 Primary Outcomes: MACE

Eight RCTs (*n* = 5071) [13, 21, 23, 24, 25, 28, 30, 34] reported 255 (5.03%) cases of MACE. The incidence of MACE was 4.34%, 5.31%, 1.78%, 15.63%, and 10.06% among patients treated with low‐dose prasugrel, low‐dose ticagrelor, standard‐dose prasugrel, standard‐dose ticagrelor, and standard‐dose clopidogrel, respectively (Table 1).

**TABLE 1:** Summary table of the results for each outcomes.

		Total			NMA included			
Outcomes	Included drugs	Studies	No. of participants	*N* (%)	Studies	No. of participants	*I* ^2^ % (pairwise and consistency)	SUCRA
MACE	Prasugrel‐low	6	2445	106 (4.34)	6	2445	0.00/0.00	0.71
	Ticagrelor‐low	3	113	6 (5.31)	3	113		0.65
	Prasugrel‐standard	3	1348	24 (1.78)	3	1348		0.36
	Ticagrelor‐standard	1	32	5 (15.63)	1	32		0.38
	Clopidogrel‐standard	4	1133	114 (10.06)	4	1133		0.39
MI^*^	Clopidogrel‐standard	5	1233	93 (7.54)	4	1133	0.00/0.00	0.29
	Prasugrel‐standard	4	1548	22 (1.42)	4	1548		0.64
	Prasugrel‐low	7	2644	92 (3.48)	7	2644		0.68
	Ticagrelor‐low	2	82	1 (1.22)	2	82		0.40
	Clopidogrel‐low	1	100	0 (0.00)	NA	NA	NA	NA
Stroke^*^	Clopidogrel‐standard	5	1172	12 (1.02)	4	1130	0.00/0.00	0.51
	Prasugrel‐low	7	2484	16 (0.64)	7	2484		0.49
	Prasugrel‐standard	3	1348	5 (0.37)	3	1348		0.50
	Ticagrelor‐low	2	82	0 (0.00)	NA	NA	NA	NA
CVD^*^	Clopidogrel‐standard	5	1233	9 (0.73)	3	1091	0.00/0.00	0.79
	Prasugrel‐low	6	2445	13 (0.53)	5	2347		0.64
	Prasugrel‐standard	3	1348	10 (0.74)	2	1253		0.07
	Clopidogrel‐low	1	100	0 (0.00)	NA	NA	NA	NA
	Ticagrelor‐low	3	144	2 (1.39)				
	Ticagrelor‐standard	1	58	2 (3.45)				
ACD^*^	Clopidogrel‐standard	4	1189	13 (1.09)	3	1089	0.00/0.00	0.69
	Prasugrel‐low	5	2362	23 (0.97)	5	2362		0.60
	Prasugrel‐standard	2	1263	15 (1.19)	2	1263		0.21
	Clopidogrel‐low	1	100	2 (2.00)	NA	NA	NA	NA
	Ticagrelor‐low	1	31	0 (0.00)				
	Ticagrelor‐standard	1	32	1 (3.13)				
Bleeding	Clopidogrel‐low	1	100	3 (3.00)	1	100	13.66/0.00	0.54
	Prasugrel‐low	4	1192	525 (44.04)	4	1192		0.59
	Ticagrelor‐low	5	254	52 (20.47)	5	254		0.58
	Ticagrelor‐standard	3	161	38 (23.60)	3	161		0.17
	Clopidogrel‐standard	6	1279	404 (31.59)	6	1279		0.90
	Prasugrel‐standard	1	95	45 (47.37)	1	95		0.21
Major bleeding^*^	Clopidogrel‐standard	5	1203	24 (2.00)	4	1161	82.79/82.24	0.09
	Prasugrel‐standard	3	1463	3 (0.21)	3	1463		0.72
	Prasugrel‐low	6	2588	17 (0.66)	6	2588		0.69
	Ticagrelor‐low	4	200	4 (2.00)	NA	NA	NA	NA
	Ticagrelor‐standard	3	147	4 (2.72)				
Minor bleeding	Ticagrelor‐low	6	291	19 (6.53)	6	291	0.00/0.00	0.46
	Prasugrel‐low	3	1953	32 (1.64)	3	1953		0.47
	Prasugrel‐standard	2	1263	6 (0.48)	2	1263		0.54
	Ticagrelor‐standard	5	239	37 (15.48)	5	239		0.86
	Clopidogrel‐standard	3	766	16 (2.09)	3	766		0.16
Minimal bleeding	Prasugrel‐low	4	1390	24 (1.73)	4	1390	0.00/0.00	0.70
	Ticagrelor‐low	4	163	30 (18.40)	4	163		0.55
	Ticagrelor‐standard	2	75	23 (30.67)	2	75		0.22
	Clopidogrel‐standard	3	129	21 (16.28)	3	129		0.74
	Prasugrel‐standard	3	1348	39 (2.89)	3	1348		0.29
Bleeding events leading to discontinuation	Prasugrel‐low	3	2225	35 (1.57)	3	2225	0.00/0.00	0.82
	Prasugrel‐standard	1	1168	35 (3.00)	1	1168		0.06
	Clopidogrel‐standard	2	1050	29 (2.76)	2	1050		0.61

Abbreviations: NA, not applicable; SUCRA, the surface under the cumulative ranking curve; MACE, major adverse cardiovascular events; CVD, cardiovascular disease; MI, myocardial infarction; ACD, all‐cause death; NMA, network meta‐analysis.

* The outcomes marked with asterisk (*) have not included all interventions in the NMA analysis, because the confidence intervals for the odds ratios were very wide when some interventions were included in the NMA analysis. Therefore, the interventions that could not be included are only presented in this table in a systematic descriptive manner.

**FIGURE 1:**
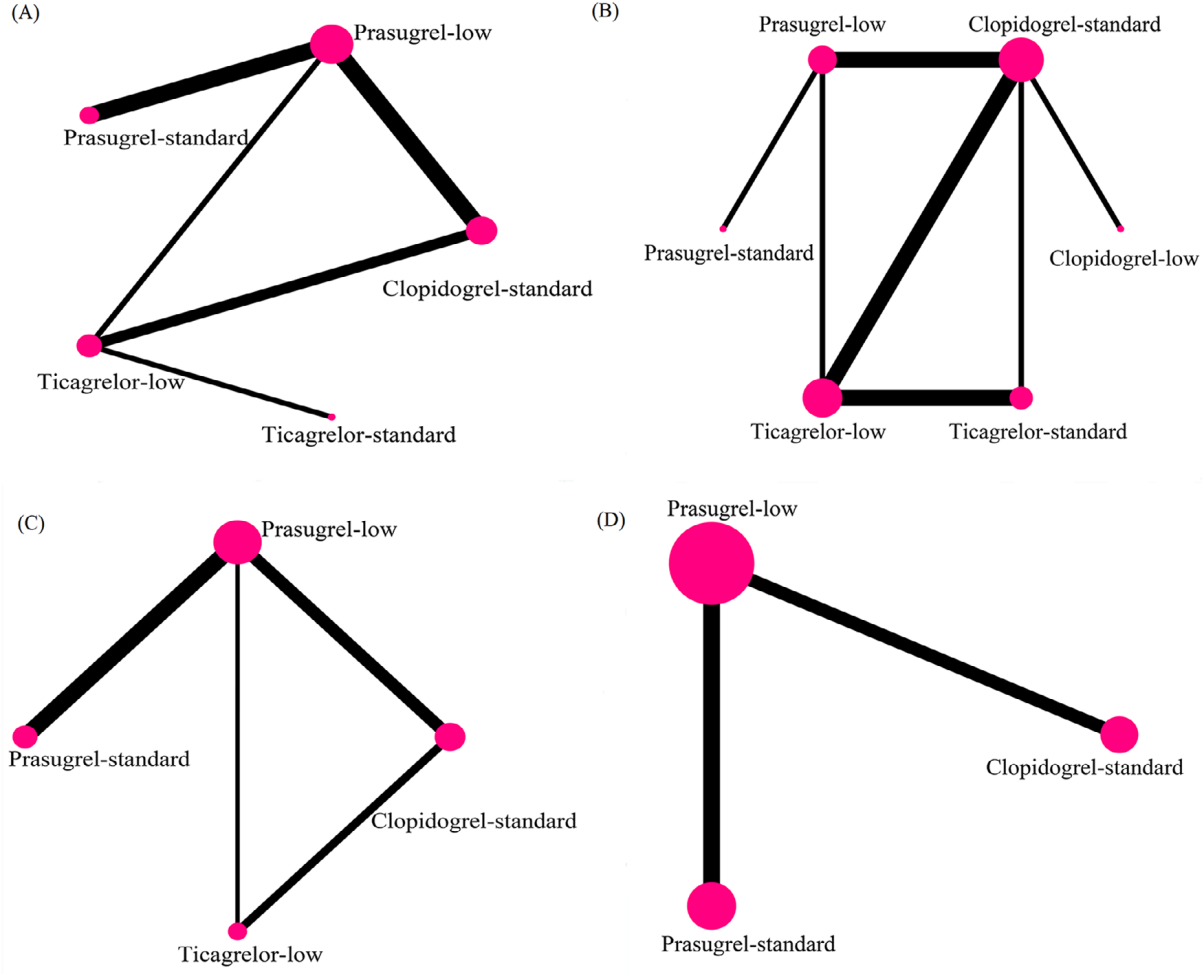
The network plot of MACE (A), bleeding (B), MI (C), and major bleeding (D). The size of the nodes of the network is proportional to the number of patients; the thickness of the connecting lines corresponds to the number of randomized controlled trials with direct comparisons. MACE, major adverse cardiovascular events; MI, myocardial infarction.

**FIGURE 2:**
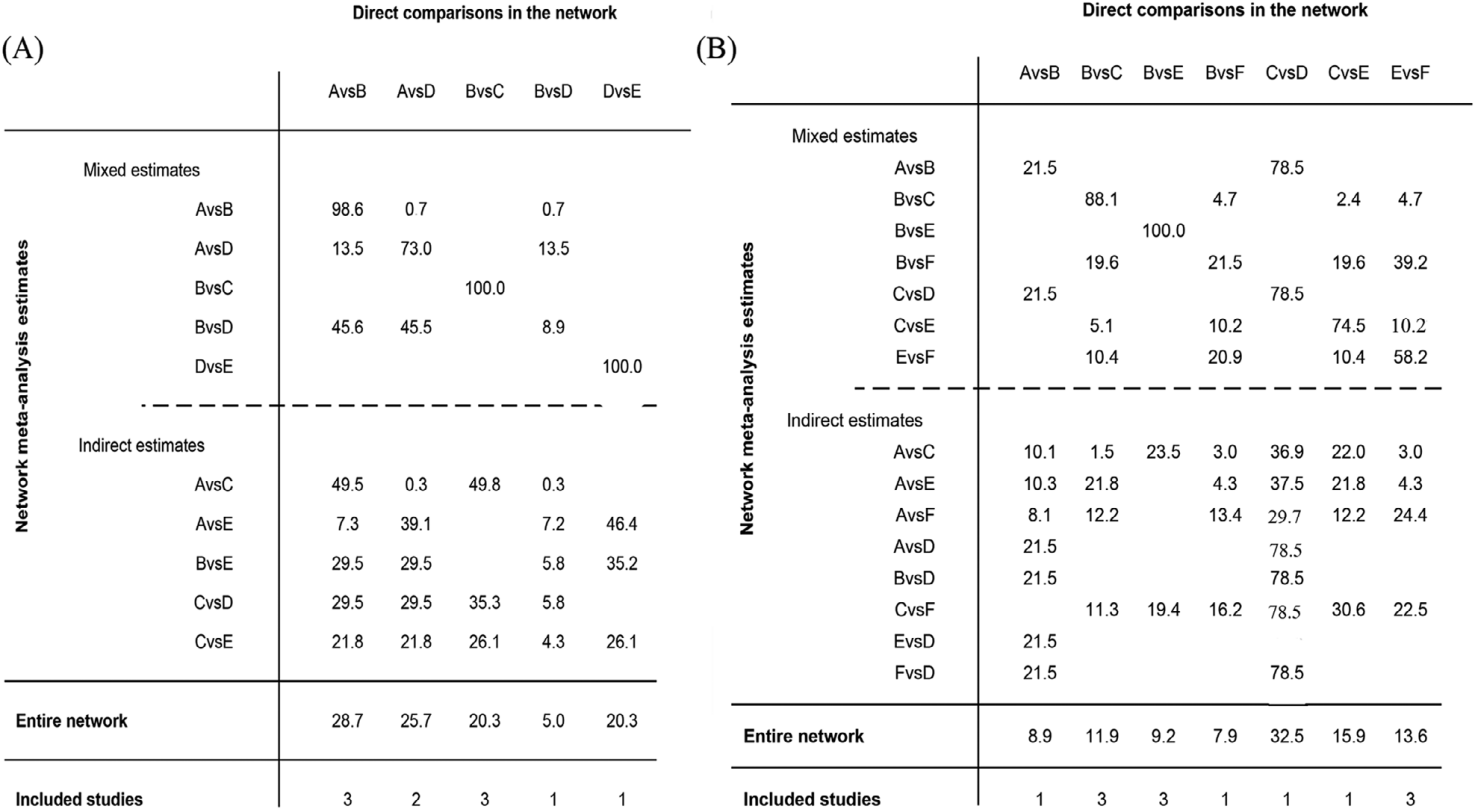
Contributions matrix plot of MACE (A) and bleeding (B). The percentage contribution of each direct estimate to the network meta‐analysis estimates. Rows correspond to network meta‐analysis (separated for mixed and indirect evidence) and columns correspond to direct meta‐analysis. The contribution of each direct comparison to the total network evidence that provides the ranking of the treatments is presented separately (row named Entire network). The last row shows the number of included direct comparisons. For example (A), 27.4 indicates the extent to which the direct comparison results of low‐dose prasugrel versus standard‐dose clopidogrel affect the entire network estimates. Similarly, 98.9 is the mixed comparison. MACE, major adverse cardiovascular events. (A): A, standard‐dose clopidogrel; B, low‐dose prasugrel; C, standard‐dose prasugrel; D, low‐dose ticagrelor; E, standard‐dose ticagrelor; F, low‐dose clopidogrel. (B): A, low‐dose clopidogrel; B, standard‐dose clopidogrel; C, low‐dose prasugrel; D, standard‐dose prasugrel; E, low‐dose ticagrelor; F, standard‐dose ticagrelor.

The network diagram was shown in Figure 1A. The contribution plots indicated that direct comparisons between low‐dose prasugrel and standard‐dose clopidogrel accounted for the largest proportion of the entire network at 28.7% (Figure 2A). Furthermore, these direct comparisons also contributed the most to the mixed estimates for both low‐dose prasugrel versus standard‐dose clopidogrel and low‐dose ticagrelor versus low‐dose prasugrel, with respective contributions of 98.6% and 45.6% (Figure 2A). The trace and density plots showed good model convergence (Figures S7.1 and S7.2). There was low heterogeneity between the studies (*I* ^2^ pairwise and consistency was 0.00% and 0.00%) (Table 1).

**FIGURE 3:**
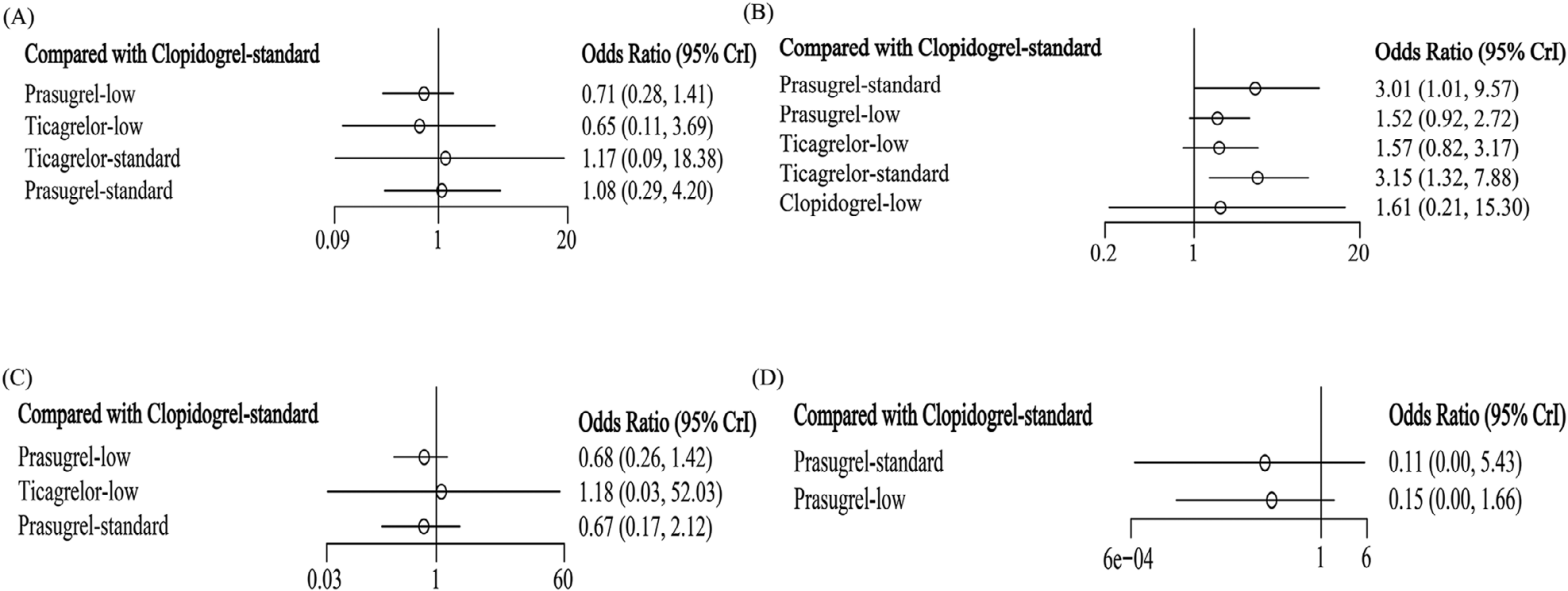
The forest plot of MACE (A), bleeding (B), MI (C), and major bleeding (D). MACE, major adverse cardiovascular events; MI, myocardial infarction.

**FIGURE 4:**
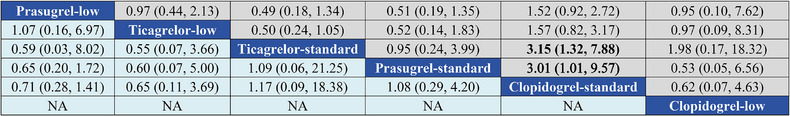
Network meta‐analysis results for major adverse cardiovascular events (light blue) and bleeding (light gray). Data are odds ratio with a 95% confidence interval. The table should be read from left to right. Reciprocals should be taken to obtain odds ratios for comparisons in the opposite direction. NA, not applicable.

**FIGURE 5:**
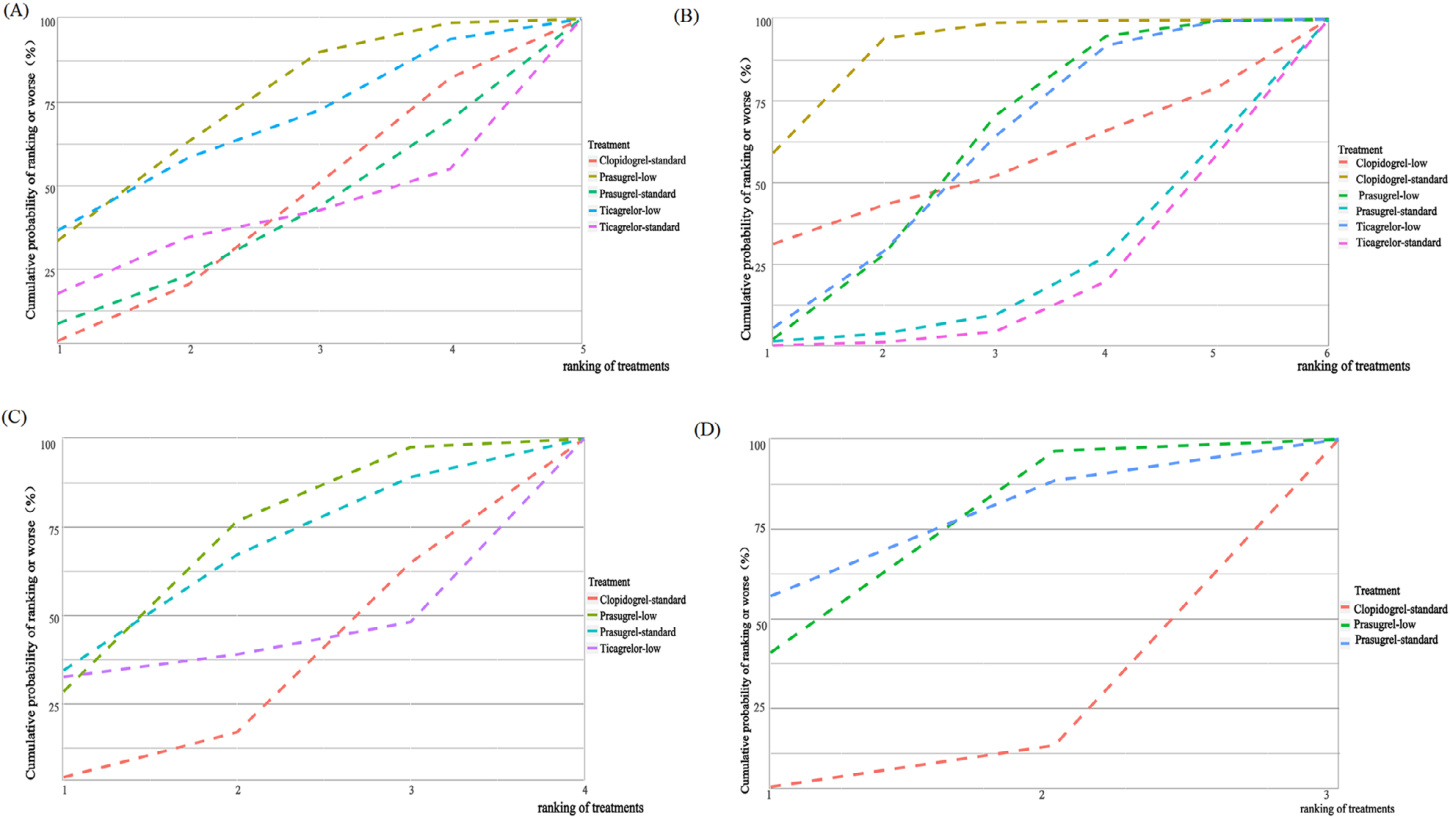
Cumulative ranking curves for MACE (A), bleeding (B), MI (C), and major bleeding (D). Graphs show the cumulative probability of each intervention ranking, from best (rank 1) to worst for each outcome. For example, low‐dose prasugrel probably ranked best for decreasing the risk of MACE. MACE, major adverse cardiovascular events; MI, myocardial infarction.

Low evidence (Table S8.1) suggested that low‐dose prasugrel (OR = 0.71, 95% CI [0.28, 1.41]), low‐dose ticagrelor (OR = 0.65, 95% CI [0.11, 3.69]), standard‐dose ticagrelor (OR = 1.17, 95% CI [0.09, 18.38]), and standard‐dose prasugrel (OR = 1.08, 95% CI [0.29, 4.20]) were comparable to standard‐dose clopidogrel in reducing the risk of MACE (Figures 3A and 4). The SUCRA values of low‐dose prasugrel, low‐dose ticagrelor, standard‐dose clopidogrel, standard‐dose ticagrelor, and standard‐dose prasugrel were 0.71, 0.65, 0.39, 0.38, and 0.36, respectively (Table 1). Low‐dose prasugrel probably ranked highest for reducing the risk of MACE (Figure 5A).

### 3.5 Primary Outcomes: Bleeding

Nine RCTs (*n* = 3081) [13, 21, 22, 23, 27, 28, 32, 33, 34] reported 1067 (34.63%) cases of bleeding. The incidence of bleeding was 44.04%, 20.47%, 3.00%, 47.37%, 23.60%, and 31.59% among patients treated with low‐dose prasugrel, low‐dose ticagrelor, low‐dose clopidogrel, standard‐dose prasugrel, standard‐dose ticagrelor, and standard‐dose clopidogrel, respectively (Table 1).

The network diagram was displayed in Figure 1B. The contribution plots showed that direct comparisons of low‐dose prasugrel versus standard‐dose prasugrel contributed the most to the entire network (32.5%) (Figure 2B). Additionally, this direct comparisons contributed to mixed estimates for low‐dose clopidogrel versus standard‐dose clopidogrel and low‐dose prasugrel versus standard‐dose prasugrel were highest at 78.5% and 78.5%, respectively (Figure 2B). The trace and density plots showed good model convergence (Figures S7.11 and S7.12). There was moderate to high heterogeneity between the studies (*I* ^2^ pairwise and consistency were 13.66% and 0.00%, respectively) (Table 1).

Low‐ to moderate‐level evidence (Table S8.6) suggested that low‐dose prasugrel (OR = 1.52, 95% CI [0.92, 2.72]), low‐dose ticagrelor (OR = 1.57, 95% CI [0.82, 3.17]), and low‐dose clopidogrel (OR = 1.61, 95% CI [0.21, 15.30]) showed similar benefits in terms of bleeding compared to standard‐dose clopidogrel (Figures 3B and 4). Standard‐dose of ticagrelor (OR = 3.15, 95% CI [1.32, 7.88]) and standard‐dose of prasugrel (OR = 3.01, 95% CI [1.01, 9.57]) increased the risk of bleeding. The SUCRA values of standard‐dose clopidogrel, low‐dose prasugrel, low‐dose ticagrelor, low‐dose clopidogrel, standard‐dose prasugrel, and standard‐dose ticagrelor were 0.90, 0.59, 0.58, 0.54, 0.21, and 0.17, respectively (Table 1). Standard‐dose clopidogrel ranked highest for reduction of bleeding (Figure 5B).

### 3.6 Other Endpoints

The studies analyzed cases of MI, ischemic stroke, CVD, ACD, major bleeding, minor bleeding, minimal bleeding, and bleeding events leading to discontinuation in both low‐dose and standard‐dose groups, as detailed in Table 1 and Supplementary Material 7–13.

Based on low‐level evidence (Tables S8.2 and S8.7), it was suggested that low‐dose ticagrelor, low‐dose prasugrel, standard‐dose prasugrel, and standard‐dose clopidogrel were consistent in reducing the risk of MI (Figures 3C and S10.1), with the latter three being consistent in increasing the risk of major bleeding (Figures 3D and S10.5). Additionally, low‐dose prasugrel and standard‐dose prasugrel probably rank highest for reducing MI and major bleeding, respectively, according to SUCRA values (Table 1, Figure 5C, D).

Both low‐dose prasugrel and standard‐dose prasugrel demonstrated similar efficacy in relation to the risk of ischemic stroke (Figures S10.2 and S11.1), CVD (Figures S10.3 and S11.2), ACD (Figures S10.4 and S11.3), minor bleeding (Figures S10.6 and S11.4), and bleeding events leading to discontinuation (Figures S10.8 and S11.6) when compared to standard‐dose clopidogrel. Additionally, low‐dose ticagrelor, low‐dose prasugrel, standard‐dose prasugrel, and standard‐dose ticagrelor present a similar risk of minor and minimal bleeding compared to standard‐dose clopidogrel (Figures S10.6, S10.7, S11.4, and S11.5).

### 3.7 Subgroup Analysis

Upon conducting a subgroup analysis on patients diagnosed with ACS, it was observed that the results for all outcomes, with the exception of minimal bleeding, consistent with the overall analysis (Supplementary Material 14). In Asian patients, the risks of ischemia and bleeding were consistent across low‐dose prasugrel, low‐dose ticagrelor, low‐dose clopidogrel, standard‐dose prasugrel, standard‐dose ticagrelor, and standard‐dose clopidogrel, as observed in the overall analysis (Supplementary Material 15). Similar results were also found in patients with a BMI range of 24–26 kg/m^2^ (Supplementary Material 16).

### 3.8 Publication Bias

Comparison‐adjusted funnel plots suggested that there might not be publication bias concerning MACE, bleeding, and minimal bleeding. However, since few studies had been included regarding MI, ischemic stroke, and other outcomes, publication bias could not be accurately reflected by a comparison‐adjusted funnel plot (Supplementary Material 17).

## 4 Discussion

This study represented the first comprehensive analysis that compared the efficacy and safety of 6 different DAPT regimens in patients with CHD, encompassing 10 outcomes related to efficacy (MACE, MI, ischemic stroke, etc.) and bleeding (bleeding, major bleeding, minor bleeding, etc.).

The main findings were as follows: (1) low‐dose of prasugrel or ticagrelor and standard‐dose ticagrelor or prasugrel or clopidogrel probably did not have different effects on MACE; (2) no difference in bleeding risk was found between low‐dose of prasugrel or ticagrelor or clopidogrel and standard‐dose of clopidogrel, ticagrelor, or prasugrel, but standard‐dose of ticagrelor or prasugrel compared to clopidogrel increased the risk of bleeding; (3) we also found no differences between low‐dose prasugrel and standard‐dose prasugrel or clopidogrel in terms of MI, ischemic stroke, ACD, CVD, major bleeding, minor bleeding, minimal bleeding, and bleeding events leading to discontinuation.

Wongsalap's meta‐analysis, which included three RCTs, showed no significant differences in terms of MACE (relative risk = 0.92, 95% CI [0.74, 1.16]) or major bleeding (relative risk = 0.97, 95% CI [0.57, 1.65]) between low‐dose prasugrel and standard‐dose clopidogrel. However, low‐dose prasugrel increased the risk of minor bleeding compared to standard‐dose clopidogrel [7]. The analysis of minor bleeding was pooled with the results of two RCTs, one of which involved elderly patients (age >75 years). Elderly individuals had a high incidence of atherosclerotic cardiovascular diseases and might exhibit intolerance to antiplatelet drugs. The GRACE registration study [36] showed that the incidence of severe bleeding events in ACS patients aged >75 years was as high as 5.3% within 1 year. This might explain the difference in minor bleeding results observed in our study.

Chen Qing's meta‐analysis, published in 2020, suggested that low‐dose ticagrelor significantly reduced MACE (OR = 0.39, 95% CI [0.26, 0.58]), increased minor or minimal bleeding events (OR = 1.64, 95% CI [1.04, 2.59]), and had similar safety regarding major bleeding (OR = 1.16, 95% CI [0.43, 3.08]) compared to standard dose clopidogrel [6]. However, these differing results might be attributed to several factors. Chen Qing's meta‐analysis included a large number of articles published in Chinese, had a relatively short follow‐up period (5 days to 12 months), and did not specify whether the intervention was DAPT.

The efficacy and safety of antiplatelet therapy varied significantly among individuals due to factors such as low reactivity of antiplatelet agents. In this study, all participants were treated with DAPT (standard‐dose aspirin plus standard‐dose or low‐dose P2Y12 receptor inhibitors). Consequently, variations in the efficacy and safety of aspirin among different patients might impact the study results. However, recent studies such as ADAPT‐DES [37] and ASCET [38] had demonstrated that low aspirin reactivity was not associated with cardiac ischemic events in patients receiving DAPT after PCI.

Although the CYP2C19 genotype was significantly associated with the prognosis of patients with CHD [39], findings from the PLATO study [40] indicated that although CYP2C19 loss‐allele carriers had significantly higher rates of primary cardiovascular endpoint events at 30 days compared with noncarriers, this difference was not statistically significant at 12 months of follow‐up in ACS patients treated with clopidogrel. Considering that the efficacy of clopidogrel in Asians was influenced by the CYP2C19 genotype, subgroup analysis of Asians also yielded consistent results.

Elderly age, uncontrolled hypertension, and renal insufficiency were known risk factors for increased bleeding from antiplatelet drugs [41, 42]. The mean age of the patients included in this study was 63.97. Interstudy heterogeneity was acceptable, with age, renal function, and combined hypertension being generally balanced among the groups in each study, except for the Kitano D'study [26]. Blood pressure control was not reported in all studies, and kidney function was not mentioned in four studies [22, 23, 27, 31]. Therefore, these confounding factors might also influence our safety outcomes.

Heterogeneity in this study was acceptable, and depending on the inclusion and exclusion criteria of this study and the characteristics of the included studies, heterogeneity might be due to patient, diagnosis, and outcome. Different types of CHD [43], race and body weight might affect the risk of ischemia and bleeding of antiplatelet drugs, subgroup analyses were conducted among patients with ACS, individuals from the Asian population, and those with varying BMIs. The findings derived from the subgroup analysis were basically consistent with those of overall analysis.

The Filippo OD study [44] also demonstrated that standard‐dose aspirin combined with low‐dose prasugrel resulted in a similar risk of MACE, MI, ACD, stroke, all bleeding events, major bleedings, and minor bleeding in patients with ACS, when compared to standard‐dose aspirin combined with standard‐dose prasugrel/clopidogrel.

The strengths of this study included a comprehensive search to identify eligible trials and independent study identification, selection, data extraction, and assessment of RoB by two reviewers. CINeMA was utilized to report the certainty of available evidence, and estimated absolute risks for all outcomes were provided. The quality of the literature included in the study was relatively high, with participants and interventions being relatively homogeneous. All participants had an average BMI of 24–29.5 kg/m^2^ and were under 70 years old, treated with aspirin plus P2Y12 receptor inhibitors. Strict definitions of low dose were applied for different disease groups, and the follow‐up time for outcomes was carefully screened to ensure optimal outcomes exposure.

There were also the following limitations in our study. Firstly, only a few studies on direct head‐to‐head comparison were included, and there was some heterogeneity in terms of bleeding and major bleeding, which resulted in a low level of evidence. Secondly, low‐dose of ticagrelor and clopidogrel were excluded from NMA due to large confidence intervals after pooled analysis, so low‐dose of ticagrelor, prasugrel and clopidogrel could not be compared in terms of other outcomes except for MACE and bleeding. Thirdly, the diversity of the definition of bleeding criteria in various studies led to the failure to combine the data of many studies. Fourthly, due to the limited data provided by the included studies, it was not feasible to conduct subgroup analyses for patients with STEMI, NSTEMI, and UA. Finally, it was crucial to consider potential biases that might have affected the study's outcomes. The geographical focused of the studies, predominantly in Asia, raised concerns about the generalizability of the findings to other parts of the world.

In summary, this NMA suggested that there was no difference in the risk of ischemia and bleeding among patients with CHD treated with low‐dose ticagrelor, prasugrel, or clopidogrel, compared to standard‐dose ticagrelor, prasugrel, or clopidogrel. However, due to the low‐level evidence, future RCTs addressing this evidence gap would be warranted.

## Conflicts of Interest

The authors declare no conflicts of interest.

## Supporting information



Supporting Information
