# Supplementary material for: Comparative Efficacy and Safety of Different Low‐Dose Platelet Inhibitors in Patients With Coronary Heart Disease: A Bayesian Network Meta‐Analysis
Source: J Evid Based Med. 2024 Dec 21;17(4):822–32. doi: 10.1111/jebm.12671 (PMC11684504; doi:10.1111/jebm.12671)
Supplement: Supplementary file 1 — Supporting Information [file JEBM-17-822-s001.docx]

**Supplementary Materials**

Content

[Supplementary Material 1: Search strategy 1](#_Toc12898)

[Supplementary Material 2: Classification of low-dose and standard-dose platelet aggregation inhibitors 9](#_Toc25041)

[Table S2.1 Classification of low-dose and standard-dose platelet aggregation inhibitors 9](#_Toc12147)

[Supplementary Material 3: Flow chart of literature screening 10](#_Toc6636)

[Figure S3.1 Flow chart of literature screening 10](#_Toc27345)

[Supplementary Material 4: Citations of included studies 11](#_Toc26593)

[Citations for the 16 eligible studies (1-16) 11](#_Toc32614)

[Supplementary Material 5: Characteristics of included studies 13](#_Toc13036)

[5.1 Characteristics of included studies 13](#_Toc30604)

[Table S5.1 Characteristics of included studies 13](#_Toc25541)

[5.2 Detailed information of included patients 18](#_Toc10687)

[Table S5.2 Detailed information of included patients 18](#_Toc19733)

[Supplementary Material 6: Risk of bias assessment 26](#_Toc947)

[6.1 The results of risk of bias assessment for each study 26](#_Toc15529)

[Table S6.1 The results of risk of bias assessment for each study 26](#_Toc32583)

[6.2 The results of risk of bias assessment for each outcome 27](#_Toc8992)

[6.2.1 MACE_RoB_chart 27](#_Toc6126)

[6.2.2 MI_RoB_chart 27](#_Toc28198)

[6.2.3 Ischemic stroke_RoB_chart 28](#_Toc20864)

[6.2.4 CVD_RoB_chart 28](#_Toc23921)

[6.2.5 ACD_RoB_chart 28](#_Toc30011)

[6.2.6 Bleeding_RoB_chart 28](#_Toc3794)

[6.2.7 Major bleeding_RoB_chart 28](#_Toc11513)

[6.2.8 Minor bleeding_RoB_chart 29](#_Toc20312)

[6.2.9 Minimal bleeding_RoB_chart 29](#_Toc549)

[6.2.10 Bleeding events leading to discontinuation or non-adherence_RoB_chart 29](#_Toc30134)

[Supplementary Material 7: Trace plot and density plot, and Brooks-Gelman-Rubin diagnosis plot 30](#_Toc9909)

[7.1 The trace and density plot of MACE 30](#_Toc9699)

[7.2 The Brooks-Gelman-Rubin diagnosis plot of MACE 31](#_Toc16986)

[7.3 The trace and density plot of MI 31](#_Toc32031)

[7.4 The Brooks-Gelman-Rubin diagnosis plot of MI 32](#_Toc16834)

[7.5 The trace and density plot of ischaemic stroke 32](#_Toc25783)

[7.6 The Brooks-Gelman-Rubin diagnosis plot of ischaemic stroke 33](#_Toc31273)

[7.7 The trace and density plot of CVD 33](#_Toc3088)

[7.8 The Brooks-Gelman-Rubin diagnosis plot of CVD 34](#_Toc14359)

[7.9 The trace and density plot of ACD 34](#_Toc17784)

[7.10 The Brooks-Gelman-Rubin diagnosis plot of ACD 35](#_Toc1251)

[7.11 The trace and density plot of bleeding 35](#_Toc31788)

[7.12 The Brooks-Gelman-Rubin diagnosis plot of bleeding 36](#_Toc28967)

[7.13 The trace and density plot of major bleeding 37](#_Toc20319)

[7.14 The Brooks-Gelman-Rubin diagnosis plot of major bleeding 37](#_Toc21547)

[7.15 The trace and density plot of minor bleeding 38](#_Toc8856)

[7.16 The Brooks-Gelman-Rubin diagnosis plot of minor bleeding 39](#_Toc12110)

[7.17 The trace and density plot of minimal bleeding 39](#_Toc13796)

[7.18 The Brooks-Gelman-Rubin diagnosis plot of minimal bleeding 40](#_Toc7659)

[7.19 The trace and density plot of bleeding events leading to discontinuation or non-adherence 41](#_Toc11436)

[7.20 The Brooks-Gelman-Rubin diagnosis plot of bleeding events leading to discontinuation or non-adherence 42](#_Toc15706)

[Supplementary Material 8: CINeMA Assessment 43](#_Toc24625)

[8.1 Confidence in effect estimates for MACE 43](#_Toc5621)

[8.2 Confidence in effect estimates for MI 43](#_Toc768)

[8.3 Confidence in effect estimates for ischaemic stroke 44](#_Toc5450)

[8.4 Confidence in effect estimates for CVD 44](#_Toc1238)

[8.5 Confidence in effect estimates for ACD 44](#_Toc27366)

[8.6 Confidence in effect estimates for bleeding 44](#_Toc25290)

[8.7 Confidence in effect estimates for major bleeding 45](#_Toc25686)

[8.8 Confidence in effect estimates for minor bleeding 45](#_Toc11710)

[8.9 Confidence in effect estimates for minimal bleeding 46](#_Toc2640)

[8.10 Confidence in effect estimates for bleeding events leading to discontinuation or non-adherence 47](#_Toc8551)

[Supplementary Material 9: Network plot 48](#_Toc4143)

[9.1 Ischaemic stroke 48](#_Toc14434)

[9.2 CVD 48](#_Toc28696)

[9.3 ACD 49](#_Toc20023)

[9.4 Minor bleeding 49](#_Toc8583)

[9.5 Minimal bleeding 50](#_Toc9788)

[9.6 Bleeding events leading to discontinuation or non-adherence 50](#_Toc1308)

[Supplementary Material 10: league table for network meta-analysis 51](#_Toc9902)

[10.1 Network meta-analysis results for MI 51](#_Toc14292)

[10.2 Network meta-analysis results for ischaemic stroke 51](#_Toc20863)

[10.3 Network meta-analysis results for CVD 51](#_Toc20761)

[10.4 Network meta-analysis results for ACD 51](#_Toc4264)

[10.5 Network meta-analysis results for major bleeding 51](#_Toc27217)

[10.6 Network meta-analysis results for minor bleeding 51](#_Toc23045)

[10.7 Network meta-analysis results for minimal bleeding 52](#_Toc973)

[10.8 Network meta-analysis results for bleeding events leading to discontinuation or non-adherence 52](#_Toc3837)

[Supplementary Material 11: Forest plot of network meta-analysis 53](#_Toc14918)

[11.1 Ischaemic stroke 53](#_Toc24201)

[11.2 CVD 53](#_Toc6488)

[11.3 ACD 53](#_Toc31370)

[11.4 Minor bleeding 53](#_Toc22038)

[11.5 Minimal bleeding 54](#_Toc7656)

[11.6 Bleeding events leading to discontinuation or non-adherence 54](#_Toc22591)

[Supplementary Material 12: Contribution plots by study outcome 55](#_Toc20550)

[12.1 Contribution plot for MACE 55](#_Toc7615)

[12.2 Contribution plot for MI 56](#_Toc21653)

[12.3 Contribution plot for ischaemic stroke 56](#_Toc21117)

[12.4 Contribution plot for CVD 57](#_Toc12539)

[12.5 Contribution plot for ACD 57](#_Toc3729)

[12.6 Contribution plot for bleeding 58](#_Toc17802)

[12.7 Contribution plot for major bleeding 58](#_Toc16545)

[12.8 Contribution plot for minor bleeding 59](#_Toc455)

[12.9 Contribution plot for minimal bleeding 59](#_Toc12230)

[12.10 Contribution plot for bleeding events leading to discontinuation or non-adherence 60](#_Toc30566)

[Supplementary Material 13: Treatment Ranking using SUCRA 61](#_Toc31197)

[13.1 Cumulative ranking curves for ischaemic stroke 61](#_Toc15332)

[13.2 Cumulative ranking curves for CVD 61](#_Toc22843)

[13.3 Cumulative ranking curves for ACD 62](#_Toc22498)

[13.4 Cumulative ranking curves for minor bleeding 62](#_Toc5399)

[13.5 Cumulative ranking curves for minimal bleeding 63](#_Toc14961)

[13.6 Cumulative ranking curves for bleeding events leading to discontinuation or non-adherence 63](#_Toc23077)

[Supplementary Material 14: Subgroup in patients with ACS 64](#_Toc6067)

[14.1 Forest for MACE in patients with ACS 64](#_Toc25916)

[14.2 Forest for MI in patients with ACS 64](#_Toc5493)

[14.3 Forest for ischaemic stroke in patients with ACS 64](#_Toc30719)

[14.4 Forest for CVD in patients with ACS 64](#_Toc32178)

[14.5 Forest for ACD in patients with ACS 65](#_Toc4055)

[14.6 Forest for bleeding in patients with ACS 65](#_Toc11809)

[14.7 Forest for major bleeding in patients with ACS 65](#_Toc22983)

[14.8 Forest for minor bleeding in patients with ACS 65](#_Toc30010)

[14.9 Forest for minimal bleeding in patients with ACS 66](#_Toc29154)

[Supplementary Material 15: Subgroup in Asia 67](#_Toc20565)

[15.1 Forest for MACE in Asia 67](#_Toc13161)

[15.2 Forest for MI in Asia 67](#_Toc23505)

[15.3 Forest for ischaemic stroke in Asia 67](#_Toc18139)

[15.4 Forest for CVD in Asia 67](#_Toc3430)

[15.5 Forest for ACD in Asia 68](#_Toc17788)

[15.6 Forest for bleeding in Asia 68](#_Toc21003)

[15.7 Forest for major bleeding in Asia 68](#_Toc17947)

[15.8 Forest for minor bleeding in Asia 68](#_Toc13343)

[15.9 Forest for minimal bleeding in Asia 69](#_Toc1324)

[Supplementary Material 16: Subgroup in patients with BMI of 24-26kg/m^2^ 70](#_Toc5036)

[16.1 Forest for MACE in patients with BMI of 24-26kg/m^2^ 70](#_Toc10852)

[16.2 Forest for MI in patients with BMI of 24-26kg/m^2^ 70](#_Toc2565)

[16.3 Forest for ischaemic stroke in patients with BMI of 24-26kg/m^2^ 70](#_Toc119)

[16.4 Forest for CVD in patients with BMI of 24-26kg/m^2^ 70](#_Toc6925)

[16.5 Forest for ACD in patients with BMI of 24-26kg/m^2^ 71](#_Toc22457)

[16.6 Forest for bleeding in patients with BMI of 24-26kg/m^2^ 71](#_Toc10719)

[16.7 Forest for major bleeding in patients with BMI of 24-26kg/m^2^ 71](#_Toc21149)

[16.8 Forest for minor bleeding in patients with BMI of 24-26kg/m^2^ 71](#_Toc5057)

[16.9 Forest for minimal bleeding in patients with BMI of 24-26kg/m^2^ 72](#_Toc4978)

[Supplementary Material 17: Comparison-adjusted funnel plots 73](#_Toc11126)

[17.1 Comparison adjusted funnel plot for MACE 73](#_Toc15569)

[17.2 Comparison adjusted funnel plot for MI 74](#_Toc24695)

[17.3 Comparison adjusted funnel plot for ischaemic stroke 74](#_Toc655)

[17.4 Comparison adjusted funnel plot for CVD 75](#_Toc30874)

[17.5 Comparison adjusted funnel plot for ACD 75](#_Toc14133)

[17.6 Comparison adjusted funnel plot for bleeding 76](#_Toc14370)

[17.7 Comparison adjusted funnel plot for major bleeding 76](#_Toc25165)

[17.8 Comparison adjusted funnel plot for minor bleeding 77](#_Toc29541)

[17.9 Comparison adjusted funnel plot for minimal bleeding 77](#_Toc7768)

[17.10 Comparison adjusted funnel plot for bleeding events leading to discontinuation or non-adherence 78](#_Toc12023)

# Supplementary Material 1: Search strategy

PubMed, Embase, the Cochrane Controlled Register of Trials and ClinicalTrials.gov, from database inception to December 27, 2022. Updated on January 29, 2024. Chinese databases including CNKI, WanFang, VIP and CBM, from database inception to October 23, 2024.

The detailed search strategy for each database was presented below:

PubMed

Participants(Coronary Disease) related

#1 "Coronary Disease"[MeSH Terms]

#2 "Coronary Artery Disease"[MeSH Terms]

#3 "Myocardial Infarction"[MeSH Terms]

#4 "Angina Pectoris"[MeSH Terms]

#5 "Myocardial Ischemia"[MeSH Terms]

#6"Coronary Heart Disease"[Title/Abstract]

#7"Coronary Heart Diseases"[Title/Abstract]

#8"atherosclerotic cardiovascular disease"[Title/Abstract]

#9"ASCVD"[Title/Abstract]

#10"coronary artery diseases"[Title/Abstract]

#11"Coronary Arteriosclerosis"[Title/Abstract]

#12"coronary atherosclerotic heart disease"[Title/Abstract]

#13"Myocardial Infarctions"[Title/Abstract]

#14"Myocardial Infarct"[Title/Abstract]

#15"Myocardial Infarcts"[Title/Abstract]

#16"Heart Attack"[Title/Abstract]

#17"Heart Attacks"[Title/Abstract]

#18"acute myocardial infarction"[Title/Abstract]

#19"acute myocardial infarctions"[Title/Abstract]

#20"stable angina"[Title/Abstract]

#21"Chronic Stable Angina"[Title/Abstract]

#22"unstable angina"[Title/Abstract]

#23"Ischemic Heart Disease"[Title/Abstract]

#24 OR #1-23

Intervention (Platelet Aggregation Inhibitors) related

#25"Platelet Aggregation Inhibitors"[MeSH Terms]

#26"Aspirin"[MeSH Terms]

#27"Clopidogrel"[MeSH Terms]

#28"ticagrelor"[MeSH Terms]

#29"Purinergic P2Y Receptor Antagonists"[MeSH Terms]

#30"Cilostazol"[MeSH Terms]

#31"Dipyridamole"[MeSH Terms]

#32"Prasugrel Hydrochloride"[MeSH Terms]

#33"Blood Platelet Aggregation Inhibitor"[Title/Abstract]

#34"Blood Platelet Aggregation Inhibitors"[Title/Abstract]

#35"Platelet Antiaggregants"[Title/Abstract]

#36"Platelet Inhibitors"[Title/Abstract]

#37"Platelet Inhibitor"[Title/Abstract]

#38"Antiplatelet Agents"[Title/Abstract]

#39"Antiplatelet Agent"[Title/Abstract]

#40"Antiplatelet Drug"[Title/Abstract]

#41"Antiplatelet Drugs"[Title/Abstract]

#42"Platelet Antagonists"[Title/Abstract]

#43"Acetylsalicylic Acid"[Title/Abstract]

#44"ASA"[Title/Abstract]

#45"Iscover"[Title/Abstract]

#46"PCR 4099"[Title/Abstract]

#47"SR 25989"[Title/Abstract]

#48"Plavix"[Title/Abstract]

#49"Clopidogrel Bisulfate"[Title/Abstract]

#50"thromboxane A2 antagonist"[Title/Abstract]

#51"Indobufen"[Title/Abstract]

#52"Ibustrin"[Title/Abstract]

#53"K 3920"[Title/Abstract]

#54"Brilique"[Title/Abstract]

#55"AZD 6140"[Title/Abstract]

#56"AZD6140"[Title/Abstract]

#57"AZD-6140"[Title/Abstract]

#58"Brilinta"[Title/Abstract]

#59"Purinergic P2Y12 Receptor Antagonists"[Title/Abstract]

#60"P2Y12 Purinoceptor Antagonist"[Title/Abstract]

#61"P2Y12 Receptor Antagonists"[Title/Abstract]

#62"P2Y12 Receptor Antagonist"[Title/Abstract]

#63"P2Y12 Purinoceptor Antagonists"[Title/Abstract]

#64"OPC 13013"[Title/Abstract]

#65"OPC-13013"[Title/Abstract]

#66"Pletal"[Title/Abstract]

#67"Dipyramidole"[Title/Abstract]

#68"sarpogrelate"[Title/Abstract]

#69"Persantine"[Title/Abstract]

#70"Persantin"[Title/Abstract]

#71"MCI 9042"[Title/Abstract]

#72"MCI-9042"[Title/Abstract]

#73"CS-747"[Title/Abstract]

#74"CS747"[Title/Abstract]

#75"Prasugrel"[Title/Abstract]

#76"LY 640315"[Title/Abstract]

#77 OR #25-76

Low dose related

#78 "low dose"[All Fields]

#79 "low-dose"[All Fields]

#80 "50mg"[All Fields]

#81 "25mg"[All Fields]

#82"50 mg twice daily"[All Fields]

#83 "5mg"[All Fields]

#84 "3.75mg"[All Fields]

#85 "60mg"[All Fields]

#86 "45 mg bid"[All Fields]

#87 "45 mg twice daily"[All Fields]

#88 OR #78-87

#89 #24 AND #77 AND #88

Studies (randomized controlled trial) related

#90

(randomized controlled trial[pt] OR controlled clinical trial[pt] OR randomized[tiab] OR placebo[tiab] OR drug therapy[sh] OR randomly[tiab] OR trial[tiab] OR groups[tiab]) NOT (animals[mh] NOT humans[mh])

#91 #89 AND #90

EMBASE

Participants (Coronary Disease) related

#1 'coronary artery disease'/de

#2 'heart infarction'/de

#3 'acute heart infarction'/de

#4 'Angina Pectoris'/de

#5 'heart muscle ischemia'/de

#6 'ischemic heart disease'/de

#7 'coronary artery atherosclerosis'/de

#8'Coronary Heart Disease':ti,ab,kw

#9'Coronary Heart Diseases':ti,ab,kw

#10'atherosclerotic cardiovascular disease':ti,ab,kw

#11'ASCVD':ti,ab,kw

#12'coronary artery diseases':ti,ab,kw

#13'Coronary Arteriosclerosis':ti,ab,kw

#14'coronary atherosclerotic heart disease':ti,ab,kw

#15 'Myocardial Infarctions':ti,ab,kw

#16'Myocardial Infarct':ti,ab,kw

#17'Myocardial Infarcts':ti,ab,kw

#18'Heart Attack':ti,ab,kw

#19'Heart Attacks':ti,ab,kw

#20'acute myocardial infarction':ti,ab,kw

#21'acute myocardial infarctions':ti,ab,kw

#22'stable angina':ti,ab,kw

#23'Chronic Stable Angina':ti,ab,kw

#24'unstable angina':ti,ab,kw

#25'Ischemic Heart Disease':ti,ab,kw

#26 OR #1-25

Intervention (Platelet Aggregation Inhibitors) related

#27 'antithrombocytic agent'/de

#28 'acetylsalicylic acid'/de

#29 'clopidogrel'/de

#30 'ticagrelor'/de

#31 'purinergic p2y receptor antagonists'/de

#32 'cilostazol'/de

#33 'dipyridamole'/de

#34 'prasugrel'/de

#35 'blood platelet aggregation inhibitor':ti,ab,kw

#36 'blood platelet aggregation inhibitors':ti,ab,kw

#37 'platelet antiaggregants':ti,ab,kw

#38 'platelet inhibitors':ti,ab,kw

#39 'platelet inhibitor':ti,ab,kw

#40 'antiplatelet agents':ti,ab,kw

#41 'antiplatelet agent':ti,ab,kw

#42 'antiplatelet drug':ti,ab,kw

#43 'antiplatelet drugs':ti,ab,kw

#44 'platelet antagonists':ti,ab,kw

#45 'acetylsalicylic acid':ti,ab,kw

#46 'iscover':ti,ab,kw

#47 'pcr 4099':ti,ab,kw

#48 'sr 25989':ti,ab,kw

#49 'plavix':ti,ab,kw

#50 'clopidogrel bisulfate':ti,ab,kw

#51 'thromboxane a2 antagonist':ti,ab,kw

#52 'indobufen':ti,ab,kw

#53 'ibustrin':ti,ab,kw

#54 'k 3920':ti,ab,kw

#55 'brilique':ti,ab,kw

#56 'azd 6140':ti,ab,kw

#57 'azd6140':ti,ab,kw

#58 'azd-6140':ti,ab,kw

#59 'brilinta':ti,ab,kw

#60 'purinergic p2y12 receptor antagonists':ti,ab,kw

#61 'p2y12 purinoceptor antagonist':ti,ab,kw

#62 'p2y12 receptor antagonists':ti,ab,kw

#63 'p2y12 receptor antagonist':ti,ab,kw

#64 'p2y12 purinoceptor antagonists':ti,ab,kw

#65 'opc 13013':ti,ab,kw

#66 'opc-13013':ti,ab,kw

#67 'pletal':ti,ab,kw

#68 'dipyramidole':ti,ab,kw

#69 'sarpogrelate':ti,ab,kw

#70 'persantine':ti,ab,kw

#71 'persantin':ti,ab,kw

#72 'mci 9042':ti,ab,kw

#73 'mci-9042':ti,ab,kw

#74 'cs-747':ti,ab,kw

#75 'cs747':ti,ab,kw

#76 'prasugrel':ti,ab,kw

#77 'ly 640315':ti,ab,kw

#78 OR #27-#77

Low dose related

#79 'low drug dose'/de

#80 'low dose'all fields

#81 'low-dose'all fields

#82 OR #79-#81

Studies (randomized controlled trial) related

#83

'crossover procedure':de OR 'double-blind procedure':de OR 'randomized controlled trial':de OR 'single-blind procedure':de OR (random* OR factorial* OR crossover* OR cross NEXT/1 over* OR placebo* OR doubl* NEAR/1 blind* OR singl* NEAR/1 blind* OR assign* OR allocat* OR volunteer*):de,ab,ti

#84 #26 AND #78 AND #82 AND #83

Cochrane Libary

Participants (Coronary Disease) related

#1 MeSH descriptor: [Coronary Disease]this term only

#2 MeSH descriptor: [Coronary Artery Disease]this term only

#3 MeSH descriptor: [Myocardial Infarction]this term only

#4 MeSH descriptor: [Angina Pectoris]this term only

#5 MeSH descriptor: [Myocardial Ischemia]this term only

#6("Coronary Heart Disease"):ti,ab,kw

#7("Coronary Heart Diseases"):ti,ab,kw

#8 ("atherosclerotic cardiovascular disease"):ti,ab,kw

#9 ("ASCVD"):ti,ab,kw

#10("coronary artery diseases"):ti,ab,kw

#11("Coronary Arteriosclerosis"):ti,ab,kw

#12("coronary atherosclerotic heart disease"):ti,ab,kw

#13("Myocardial Infarctions"):ti,ab,kw

#14("Myocardial Infarct"):ti,ab,kw

#15("Myocardial Infarcts"):ti,ab,kw

#16("Heart Attack"):ti,ab,kw

#17("Heart Attacks"):ti,ab,kw

#18("acute myocardial infarction"):ti,ab,kw

#19("acute myocardial infarctions"):ti,ab,kw

#20("stable angina"):ti,ab,kw

#21("Chronic Stable Angina"):ti,ab,kw

#22("unstable angina"):ti,ab,kw

#23("Ischemic Heart Disease"):ti,ab,kw

#24 OR #1-23

Intervention (Platelet Aggregation Inhibitors) related

#25MeSH descriptor: [Platelet Aggregation Inhibitors]this term only

#26MeSH descriptor: [Aspirin]this term only

#27MeSH descriptor: [Clopidogrel]this term only

#28MeSH descriptor: [ticagrelor]this term only

#29MeSH descriptor: [Purinergic P2Y Receptor Antagonists]this term only

#30MeSH descriptor: [Cilostazol]this term only

#31MeSH descriptor: [Dipyridamole]this term only

#32MeSH descriptor: [Prasugrel Hydrochloride]this term only

#33("Blood Platelet Aggregation Inhibitor"):ti,ab,kw

#34("Blood Platelet Aggregation Inhibitors"):ti,ab,kw

#35("Platelet Antiaggregants"):ti,ab,kw

#36("Platelet Inhibitors"):ti,ab,kw

#37("Platelet Inhibitor"):ti,ab,kw

#38("Antiplatelet Agents"):ti,ab,kw

#39("Antiplatelet Agent"):ti,ab,kw

#40("Antiplatelet Drug"):ti,ab,kw

#41("Antiplatelet Drugs"):ti,ab,kw

#42("Platelet Antagonists"):ti,ab,kw

#43("Acetylsalicylic Acid"):ti,ab,kw

#44("ASA"):ti,ab,kw

#45("Iscover"):ti,ab,kw

#46("PCR 4099"):ti,ab,kw

#47("SR 25989"):ti,ab,kw

#48("Plavix"):ti,ab,kw

#49("Clopidogrel Bisulfate"):ti,ab,kw

#50("thromboxane A2 antagonist"):ti,ab,kw

#51("Indobufen"):ti,ab,kw

#52("Ibustrin"):ti,ab,kw

#53("K 3920"):ti,ab,kw

#54("Brilique"):ti,ab,kw

#55("AZD 6140"):ti,ab,kw

#56("AZD6140"):ti,ab,kw

#57("AZD-6140"):ti,ab,kw

#58("Brilinta"):ti,ab,kw

#59("Purinergic P2Y12 Receptor Antagonists"):ti,ab,kw

#60 ("P2Y12 Purinoceptor Antagonist"):ti,ab,kw

#61("P2Y12 Receptor Antagonists"):ti,ab,kw

#62("P2Y12 Receptor Antagonist"):ti,ab,kw

#63("P2Y12 Purinoceptor Antagonists"):ti,ab,kw

#64("OPC 13013"):ti,ab,kw

#65("OPC-13013"):ti,ab,kw

#66("Pletal"):ti,ab,kw

#67("Dipyramidole"):ti,ab,kw

#68("sarpogrelate"):ti,ab,kw

#69("Persantine"):ti,ab,kw

#70("Persantin"):ti,ab,kw

#71("MCI 9042"):ti,ab,kw

#72 ("MCI-9042"):ti,ab,kw

#73("CS-747"):ti,ab,kw

#74("CS747"):ti,ab,kw

#75("Prasugrel"):ti,ab,kw

#76("LY 640315"):ti,ab,kw

#77 OR #25-76

Low dose related

#78 "low dose"[All Text]

#79 "low-dose"[All Text]

#80 "50mg"[All Text]

#81 "25mg"[All Text]

#82"50 mg twice daily"[All Text]

#83 "5mg"[All Text]

#84 "3.75mg"[All Text]

#85 "60mg"[All Text]

#86 "45 mg bid"[All Text]

#87 "45 mg twice daily"[All Text]

#88 OR #78-87

#89 #24 AND #77 AND #88

Studies (randomized controlled trial) related

#90 MeSH descriptor: [Randomized Controlled Trials as Topic] this term only

#91 ("randomized controlled trial"):ti,ab,kw

#92 ("randomized control trial"):ti,ab,kw

#93 ("randomized controlled trials"):ti,ab,kw

#94 ("rct"):ti,ab,kw

#95 ("rcts"):ti,ab,kw

#96 ("randomized"):ti,ab,kw

#97 ("placebo"):ti,ab,kw

#98 ("randomly"):ti,ab,kw

#99 OR #90-98

#100 #89 AND #99

ClinicalTrials.gov

#1 Condition or disease: Coronary disease

#2 Study Results: Studies With results

#3 Intervention/treatment: Platelet aggregation inhibitors

CNKI, WanFang, VIP and CBM

The following are the corresponding English translations of the Chinese subject terms：

Participants (Coronary Disease) related

Coronary Disease

Myocardial Infarction

ASCVD

Angina Pectoris

Ischemic Heart Disease

Intervention (Platelet Aggregation Inhibitors) related

Aspirin

Clopidogrel

ticagrelor

Cilostazol

Prasugrel

Indobufen

Antiplatelet Agents

Dipyridamole

Blood Platelet Aggregation Inhibitor

Platelet Aggregation Inhibitors

Studies (randomized controlled trial) related

randomized controlled trial

RCT

RCTs

Low dose related

Low dose

# Supplementary Material 2: Classification of low-dose and standard-dose platelet aggregation inhibitors

**Table S2.1 Classification of low-dose and standard-dose platelet aggregation inhibitors**

| **Platelet aggregation inhibitors** | **Standard dose** | **Low dose** |
| --- | --- | --- |
| Aspirin | 100mg QD | 50mg QD, 25mg QD |
| Indobufen | 100-200mg BID | 100mg QD |
|  | Age>65: 100mg QD | NA |
| Clopidogrel | 75mg QD | 50mg QD, 25mg QD, 25mg BID |
| Prasugrel | Weight≥60 kg and age<75: maintenance dose: 10mg QD | 5mg QD, 3.75mg QD |
|  | Weight<60 kg:maintenance dose:5 mg once daily | 3.75mg |
|  | Age>75:maintenance dose: 5 mg once daily | 3.75mg |
| Ticagrelor | Maintenance dose:90mg BID | 60mg BID, 60mg QD, 45mg BID |
|  | Patients had a history of myocardial infarction for at least 1 year with at least one high risk factor for atherothrombotic events:60mg BID | 60mg QD, 45mg BID |
|  | In patients with ACS who are at high risk for atherothrombotic events, continuous therapy with 60mg twice daily may be started immediately after 1 year of treatment with 90 mg of acridine or another adenosine diphosphate receptor inhibitor | 60mg QD, 45mg BID |
| Cilostazol | 0.1g BID | 50mg BID, 50mg QD, 100mg QD |
| Dipyridamole | 25-50mg TID | 50mg QD, 25mg QD, 25mg BID |

Classification is based on manuals and literatures. NA, not applicable; ACS, acute coronary syndrome; QD, quaque die; BID, bis in die; TID, ter in die.

# Supplementary Material 3: Flow chart of literature screening


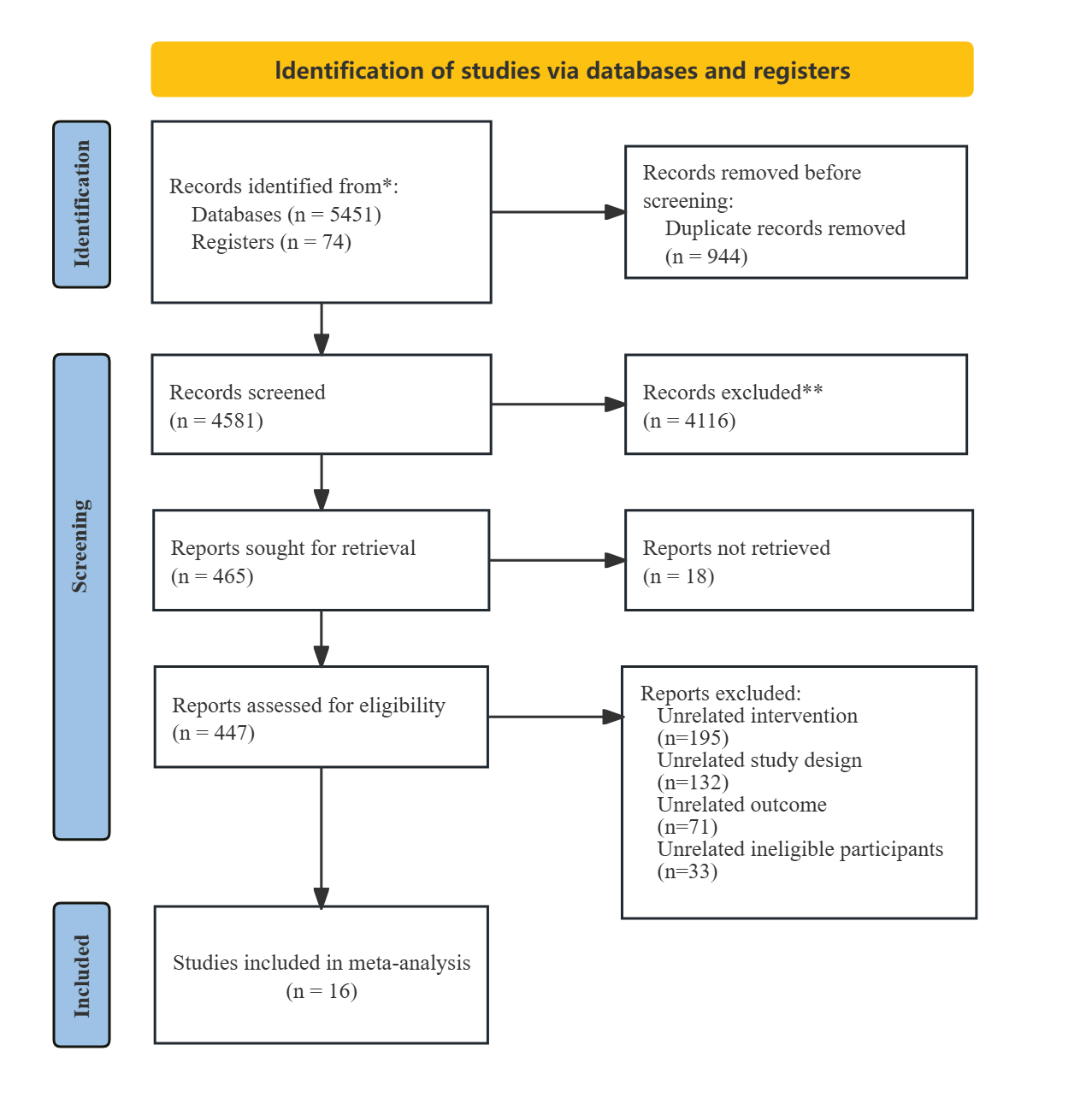


## Figure S3.1 Flow chart of literature screening

Supplementary Material 4: Citations of included studies

## Citations for the 16 eligible studies (1-16)

1. Wang Y, Jiang Y, Zhi W, Fu Y, Wang Q, Zhou J, et al. Safety and feasibility of low-dose ticagrelor in patients with ST-segment elevation myocardial infarction. Clin Cardiol. 2021;44(1):123-8.

2. Jeong YH, Oh JH, Yoon HJ, Park Y, Suh J, Lee SW, et al. Pharmacodynamic Profile and Prevalence of Bleeding Episode in East Asian Patients with Acute Coronary Syndromes Treated with Prasugrel Standard-Dose versus De-escalation Strategy: A Randomized A-MATCH Trial. Thromb Haemost. 2021;121(10):1376-86.

3. Kitano D, Takayama T, Fukamachi D, Migita S, Morikawa T, Tamaki T, et al. Impact of low-dose prasugrel on platelet reactivity and cardiac dysfunction in acute coronary syndrome patients requiring primary drug-eluting stent implantation: A randomized comparative study. Catheter Cardiovasc Interv. 2020;95(1):E8-E16.

4. Kim HS, Kang J, Hwang D, Han JK, Yang HM, Kang HJ, et al. Prasugrel-based de-escalation of dual antiplatelet therapy after percutaneous coronary intervention in patients with acute coronary syndrome (HOST-REDUCE-POLYTECH-ACS): an open-label, multicentre, non-inferiority randomised trial. Lancet. 2020;396(10257):1079-89.

5. Ueno T, Koiwaya H, Sasaki KI, Katsuki Y, Katsuda Y, Murasato Y, et al. Changes in P2Y12 reaction units after switching treatments from prasugrel to clopidogrel in Japanese patients with acute coronary syndrome followed by elective coronary stenting. Cardiovasc Interv Ther. 2017;32(4):341-50.

6. Jin CD, Kim MH, Bang J, Serebruany V. A Prospective, Randomized, Open-Label, Blinded, Endpoint Study Exploring Platelet Response to Half-Dose Prasugrel and Ticagrelor in Patients with the Acute Coronary Syndrome: HOPE-TAILOR Study. Cardiology. 2017;138(4):201-6.

7. Carrabba N, Parodi G, Marcucci R, Valenti R, Gori AM, Migliorini A, et al. Bleeding events and maintenance dose of prasugrel: BLESS pilot study. Open Heart. 2016;3(2):e000460.

8. Saito S, Isshiki T, Kimura T, Ogawa H, Yokoi H, Nanto S, et al. Efficacy and safety of adjusted-dose prasugrel compared with clopidogrel in Japanese patients with acute coronary syndrome: the PRASFIT-ACS study. Circ J. 2014;78(7):1684-92.

9. Ohkubo K, Fujimoto Y, Iwata Y, Kitahara H, Kadohira T, Sugimoto K, et al. Efficacy and safety of low-dose clopidogrel in Japanese patients after drug-eluting stent implantation: a randomized pilot trial. Heart Vessels. 2014;29(1):1-6.

10. Isshiki T, Kimura T, Ogawa H, Yokoi H, Nanto S, Takayama M, et al. Prasugrel, a third-generation P2Y12 receptor antagonist, in patients with coronary artery disease undergoing elective percutaneous coronary intervention. Circ J. 2014;78(12):2926-34.

11. Hiasa Y, Teng R, Emanuelsson H. Pharmacodynamics, pharmacokinetics and safety of ticagrelor in Asian patients with stable coronary artery disease. Cardiovascular Intervention and Therapeutics. 2014;29(4):324-33.

12. Wiviott SD, Antman EM, Winters KJ, Weerakkody G, Murphy SA, Behounek BD, et al. Randomized comparison of prasugrel (CS-747, LY640315), a novel thienopyridine P2Y12 antagonist, with clopidogrel in percutaneous coronary intervention: results of the Joint Utilization of Medications to Block Platelets Optimally (JUMBO)-TIMI 26 trial. Circulation. 2005;111(25):3366-73.

13. Hui. W, Ning. Y, Yingwu. L, Yuming L. Efficacy and safty of different dose of ticagrelor in elderly women with acute myocardial infarction after primary PCI. Chin J Geriatr Heart Brain Vessel Dis. 2022;24(9).

14. Zhiping. L, Yimin. L, Danxin. M, Chang. D, Jin H. Effect of low dose ticagrelor on coronary microcirculation, MHR, and NLR in peripheral blood of patients with stable coronary heart disease. Translational Medicine Journal. 2022;11(3):152-156.

15. Junwei. W, Liang. L, Junhua. Z, Xuan G. Effect of Low-Dose Ticagrelor Combined with Aspirin on the Efficacy in Elderly Patients after Percutaneous Coronary Intervention. Beijing Medical Journal. 2021;43(6):578-581.

16. Junnan. Y, Tingting D. Efficacy of low-dose Ticagrelor in treatment of patients with unstable angina pectoris. Zhejiang Medical Journal. 2021;43(22):2465-2467,2479.

Supplementary Material 5: Characteristics of included studies

## 5.1 **Characteristics of included studies**

**Table S5.1 Characteristics of included studies**

| **Study** | **Trial registration** | **Country** | **Population (%)** | **Duration** | **Outcome** | **No of**  **patients**  **randomized** | **Treatment** | **Drug class** | **Age** | **Female (%)** | **Mean**  **BMI**  **(kg/m^2^)** |
| --- | --- | --- | --- | --- | --- | --- | --- | --- | --- | --- | --- |
| Jeong YH 2021 | NCT01951001 | Korea | **ACS:**  UA (21.7)  NSTEMI (36.1)  NSTEMI (42.2) | 1 year | MACE/MI/Ischaemic stroke/CVD/Minimal bleeding/Fatal bleeding | 83 | prasugrel 5mg QD+aspirin 100mg QD | low | 57.40 | 11.00 | 25.40 |
|  |  |  | **ACS:**  UA (28.2)  NSTEMI (31.8)  NSTEMI (40.0) |  |  | 85 | prasugrel 10mg QD+aspirin 100mg QD | standard | 55.10 | 6.00 | 25.70 |
| Wang Y 2021 | NA | China | **ACS:**  STEMI (100) | 6 months | MACE/ACD/Minor bleeding/Minimal bleeding/ | 31 | ticagrelor 45mg BID+aspirin 100mg QD | low | 55.65 | 5.00 | 25.97 |
|  |  |  |  |  |  | 32 | ticagrelor 90mg BID+aspirin 100mg QD | standard | 55.44 | 5.00 | 25.60 |
| Kim HS 2020 | NCT02193971 | Korea | **ACS:**  STEMI (14.8)  NSTEMI (26.3)  UA (58.9) | 1 year | MI/Ischaemic stroke/CVD/ACD/Major bleeding/Minor bleeding/Minimal bleeding/Bleeding events leading to discontinuation | 1170 | prasugrel 5mg QD+aspirin 100mg QD | low | 58.70 | 10.30 | 25.70 |
|  |  |  | **ACS**:  STEMI (58.9)  NSTEMI (24.2)  UA (62.7) |  |  | 1168 | prasugrel 10mg QD+aspirin 100mg QD | standard | 58.90 | 11.20 | 25.80 |
| Jin C 2021 | NCT02944123 | Korean | **ACS**:  UA (15.4)  NSTEMI (15.4)  STEMI (59.0) | 3years | MACE/MI/Ischaemic stroke/CVD/Bleeding/Minimal bleeding/ | 39 | prasugrel 5 mg QD+aspirin 100mg QD | low | 57.00 | 2.00 | 25.00 |
|  |  |  | **ACS**:  UA (7.5)  NSTEMI (25.0)  STEMI (67.5) |  |  | 40 | ticagrelor 45mg BID+aspirin 100mg QD | low | 61.00 | 6.00 | 24.60 |
|  |  |  | **ACS:**  UA (56.1)  NSTEMI (29.3)  STEMI (14.6) |  |  | 41 | clopidogrel 75mg QD+aspirin 100mg QD | standard | 63.00 | 3.00 | 24.90 |
| Ueno T 2017 | UMIN000015122 | Japan | **ACS** (48.5):  UA (28.1)  NSTEMI (18.8)  STEMI (53.1)  **Non-ACS** (51.5) | 6 weeks | Major bleeding | 66 | prasugrel 3.75mg QD+aspirin 81–100mg QD | low | 68.30 | 14.00 | NR |
|  |  |  | **ACS** (46.2):  UA (50.0)  NSTEMI (6.7)  STEMI (43.3)  **Non-ACS** (53.8) |  |  | 65 | clopidogrel 75mg QD+aspirin 81–100 mg QD | standard | 69.30 | 18.00 | NR |
| Carrabba N 2016 | NCT01790854 | Italy | **ACS** (100) | 1 year | MACE/MI/Ischaemic stroke/CVD/ACD/Bleeding/Major bleeding/Minor bleeding/Minimal bleeding | 98 | prasugrel 5 mg QD+ aspirin 100mg QD | low | 62.20 | 14.30 | 27.20 |
|  |  |  |  |  |  | 95 | prasugrel 10 mg QD+ aspirin 100mg QD | standard | 62.20 | 13.70 | 27.50 |
| Isshiki T 2014 | JapicCTI-111550 | Japan | **ACS:**  Stable angina (74.9)  Prior MI (5.7)  UA (8.1)  Silent myocardial ischemia (11.1) | 24-48weeks | MACE/MI/Ischaemic stroke/CVD/ACD/Bleeding/Major bleeding/Major and minor bleeding/Bleeding events leading to discontinuation | 370 | prasugrel 3.75mg QD+aspirin 81–100mg QD | low | 67.50 | 25.90 | 24.49 |
|  |  |  | **ACS:**  Stable angina (76.3)  Prior MI (4.3)  UA (9.4)  Silent myocardial ischemia (8.9) |  |  | 372 | clopidogrel 75mg QD+aspirin 81–100mg QD | standard | 67.40 | 29.30 | 24.64 |
| Ohkubo K 2014 | NA | Japan | UA (29.0)  Stable angina (43.0)  Silent ischemia (28.0) | 12 months | MI/CVD/ACD/Bleeding/ | 100 | clopidogrel 50mg QD +aspirin 100mg QD | low | 66.20 | 24.00 | 24.20 |
|  |  |  | UA (36.0)  Stable angina (47.0)  Silent ischemia (17.0) |  |  | 100 | clopidogrel 75mg QD +aspirin 100mg QD | standard | 64.80 | 22.00 | 25.10 |
| Saito S 2014 | JapicCTI-101339 | Japan | **ACS:**  UA/NSTEMI (50.1)  UA (22.8)  NSTEMI (27.3)  STEMI (49.6) | 24–48 weeks | MACE/MI/Ischaemic stroke/CVD/ACD/Bleeding/Major bleeding/Minor bleeding/Major and minor bleeding/Fatal bleeding/Bleeding events leading to discontinuation | 685 | prasugrel 3.75mg QD +aspirin 81–100mg QD | low | 65.40 | 21.80 | 24.20 |
|  |  |  | **ACS:**  UA/NSTEMI (49.7)  UA (18.3)  NSTEMI (31.4)  STEMI (50.3) |  |  | 678 | clopidogrel 75mg QD +aspirin 81–100mg QD | standard | 65.10 | 20.60 | 24.20 |
| Hiasa Y 2014 | NCT01118325 | Japan and the Philippines | **CCS** (100) | 4 weeks | Bleeding/Major bleeding/Minor bleeding/Minimal bleeding | 50 | ticagrelor 45 mg BID+ aspirin 75–100 mg QD | low | 63.00 | 4.00 | 24.90 |
|  |  |  |  |  |  | 43 | ticagrelor 90 mg BID+aspirin 75–100 mg QD | standard | 64.00 | 3.00 | 24.80 |
|  |  |  |  |  |  | 46 | clopidogrel 75 mg QD+aspirin 75–100 mg QD | standard | 64.00 | 8.00 | 25.20 |
| Kitano D 2020 | UMIN000015192 | Japan | **ACS:**  STEMI (43.6)  NSTEMI (28.2)  UA (28.2) | 12months | Ischaemic stroke/ACD | 39 | prasugrel 3.75mg QD+aspirin 100mg QD | low | 65.60 | 69.00 | 24.50 |
|  |  |  | **ACS:**  STEMI (53.8)  NSTEMI (28.2)  UA (17.9) |  |  | 39 | clopidogrel 75mg QD+aspirin 100mg QD | standard | 64.00 | 67.00 | 24.50 |
| Wiviott SD 2005 | NA | United States and Canada | elective or urgent PCI:  NSTE-ACS (40) | 30 days | MI/Major bleeding/Major and minor bleeding | 199 | prasugrel 7.5mg QD+aspirin 325mg QD | low | NA | 24.00 | 29.40 |
|  |  |  |  |  |  | 200 | prasugrel 10mg QD+aspirin 325mg QD | standard | 59.00 | 25.00 | 29.50 |
| Wang H 2022 | NA | China | ACS: STEMI (100) | 1 year | CVD/Bleeding/Major bleeding/Minor bleeding | 62 | ticagrelor 45 mg BID+ aspirin 100mg QD | low | 75.60 | 100.00 | 23.40 |
|  |  |  |  |  |  | 58 | ticagrelor 90 mg BID+ aspirin 100mg QD | standard | 74.30 | 100.00 | 22.70 |
| Lu ZP 2022 | NA | China | CCS (100) | 6 months | Major bleeding/Minor bleeding | 46 | ticagrelor 60 mg BID+ aspirin 100mg QD | low | 54.71 | 45.65 | 23.85 |
|  |  |  |  |  |  | 46 | ticagrelor 90 mg BID+ aspirin 100mg QD | standard | 55.42 | 47.83 | 24.22 |
| Wang JW 2021 | NA | China | ACS/CCS | 1 year | MACE/MI/Ischaemic stroke/CVD/Bleeding/Major bleeding/Minor bleeding//Minimal bleeding | 42 | ticagrelor 60 mg BID+ aspirin 100mg QD | low | 74.40 | 54.76 | 23.70 |
|  |  |  |  |  |  | 42 | clopidogrel 75mg QD+aspirin 100mg QD | standard | 78.40 | 59.52 | 23.20 |
| Ye JN 2021 | NA | China | UA (100) | 6 months | Bleeding/Minor bleeding | 60 | ticagrelor 60 mg BID+ aspirin 100mg QD | low | 68.74 | 45.00 | NA |
|  |  |  |  |  |  | 60 | ticagrelor 90 mg BID+ aspirin 100mg QD | standard | 69.26 | 25.00 | NA |

ACS, acute coronary syndrome; UA, unstable angina; MACE, mjor adverse cardiovascular events; STEMI, ST-segment elevation myocardial infarction; NSTEMI, non-ST-elevation myocardial infarction; NSTE-ACS, non-ST-elevation acute coronary syndrome; CCS, chronic coronary syndrome; MI, myocardial infarction; PCI, percutaneous coronary intervention; Non-ACS, non-acute coronary syndrome; CVD, cardiovascular disease; NA, not applicable.

## 5.2 Detailed information of included patients

**Table S5.2 Detailed information of included patients**

| **Study Author** | **Treatment** | **Comorbidities (%)** | **Concomitant Medications (%)** | **Smoker (%)** |
| --- | --- | --- | --- | --- |
| Jeong YH 2021 | prasugrel 5mg QD+aspirin 100mg QD | Hypertension (60.20)  DM (22.90)  Dyslipidemia (79.50)  CKD (12.00)  Previous MI (4.80)  Previous PCI (3.60) | Aspirin (100.00)  β-blocker (79.50)  ACEI/ARB (68.70)  Statin (100.00)  Calcium channel blocker (13.30)  PPI (13.30) | 44.60 |
|  | prasugrel 10mg QD+aspirin 100mg QD | Hypertension (57.60)  DM (25.90)  Dyslipidemia (82.40)  CKD (3.50)  Previous MI (3.50)  Previous PCI (7.10) | Aspirin (100.0)  β-blocker (68.20)  ACEI/ARB (71.80)  Statin (98.80)  Calcium channel blocker (15.30)  PPI (22.40) | 51.80 |
| Wang Y 2021 | ticagrelor 45mg BID+aspirin 100mg QD | Hypertension (61.30)  Diabetes (25.80)  Stroke (6.50)  Anterior wall MI (54.80) | Aspirin (100.00)  Statins (93.50)  β-blocker (80.60)  ACEI/ARB (77.40) | NA |
|  | ticagrelor 90mg BID+aspirin 100mg QD | Hypertension (56.30)  Diabetes (28.10)  Stroke (9.40)  Anterior wall MI (59.40) | Aspirin (96.90)  Statins (96.90)  β-blocker (78.10)  ACEI/ARB (78.10) | NA |
| Kim HS 2020 | prasugrel 5mg QD+aspirin 100mg QD | Hypertension (62.60)  Diabetes (43.80)  Dyslipidaemia (76.10)  CKD (2.60)  PVD (1.70)  Previous MI (3.00)  Previous revascularisation (9.70)  Previous stroke(1.20) | Aspirin (98.90)  β-blocker (56.50)  ACEI/ARB (58.40)  Calcium channel blocker (22.70)  PPI (18.90)  Statin (95.30) | 59.70 |
|  | prasugrel 10mg QD+aspirin 100mg QD | Hypertension (63.60)  Diabetes(40.90)  Dyslipidaemia (77.80)  CKD (2.90)  PVD (0.80)  Previous MI (4.70)  Previous revascularisation (12.70)  Previous stroke (1.50) | Aspirin 1150/1160 (99.10)  β-blocker (53.80)  ACEI/ARB (55.50)  Calcium channel blocker (20.80)  PPI (17.20)  Statin (94.30) | 56.20 |
| Jin C 2021 | prasugrel 5mg QD+aspirin 100mg QD | DM (15.40)  Hypertension (33.30)  Dyslipidemia (17.90)  Previous MI (10.30)  Previous PCI (12.80) | Aspirin (100.00)  ACEI/ARB (23.10)  β-blocker (76.90)  Statin (94.90), PPI (20.50)  Calcium channel blocker (23.10) | 20.50 |
|  | ticagrelor 45mg BID+aspirin 100mg QD | DM (22.50)  Hypertension (52.50)  Dyslipidemia (25.00)  Previous MI (10.00)  Previous PCI (20.00) | Aspirin (100.00)  ACEI/ARB (22.50)  β-blocker (75.00)  Statin (95.00), PPI (25.00)  Calcium channel blocker (23.10) | 22.50 |
|  | clopidogrel 75mg QD+aspirin 100mg QD | DM (34.10)  Hypertension (43.90)  Dyslipidemia (12.20)  Previous MI (14.60)  Previous PCI (22.00) | Aspirin (100.00)  ACEI/ARB (29.30)  β-blocker (68.30)  Statin (92.70)  Calcium channel blocker (26.80)  PPI (17.10) | 24.40 |
| Ueno T 2017 | prasugrel 3.75mg QD+aspirin 81–100mg QD | Hypertension (72.70)  Dyslipidemia (72.70)  DM (40.90)  Hepatic function disorder (9.10)  Renal function disorder (7.60)  MI (3.0), UA (3.00)  Ischemic stroke (0.00)  Hemorrhage intracranial (0.00)  Peripheral arterial disease (3.00)  Stable angina pectoris (7.60) | Calcium antagonist (43.90)  ACEI (4.50)  Angiotensin II receptor antagonist (37.90)  β-blocker (9.10)  Statin (47.00)  Insulin (12.10)  PPI (30.30) | NA |
|  | clopidogrel 75mg QD+aspirin 81–100 mg QD | Hypertension (63.10)  Dyslipidemia (81.50)  DM (40.00)  Hepatic function disorder (3.10)  Renal function disorder (3.10)  MI (9.20), UA (3.10)  Ischemic stroke (0.00)  Hemorrhage intracranial (1.50)  Peripheral arterial disease (4.60)  Stable angina pectoris (4.60) | Calcium antagonist (38.50)  ACEI (7.70)  Angiotensin II receptor antagonist (26.20)  β-blockerr (13.80)  Statin (13.80)  Insulin (4.60)  PPI (36.90) | NA |
| Carrabba N 2016 | prasugrel 5mg QD+ aspirin 100mg QD | DM (21.40)  Hyperlipidaemia (43.90)  Hypertension (57.10)  Previous MI (17.30)  Previous PCI (28.60)  Chronic renal failure (4.10)  STEMI (27.60) | Aspirin (100.00)  Statins (93.90)  ACEI//ARB (79.60)  β-blockers (61.20)  PPIs (72.40) | 39.80 |
|  | prasugrel 10mg QD+ aspirin 100mg QD | DM (30.50)  Hyperlipidaemia (50.50)  Hypertension (57.90)  Previous MI (25.30)  Previous PCI (33.70)  Chronic renal failure (8.60)  STEMI (30.50) | Aspirin (100.00)  Statins (96.80)  ACEI//ARB (70.50)  β-blocker (73.70)  PPIs (82.10) | 30.50 |
| Isshiki T 2014 | prasugrel 3.75mg QD+aspirin 81–100mg QD | Hypertension (79.70)  Dyslipidemia (80.00)  Diabetes (40.50)  Prior ischemic stroke (3.80)  Prior transient ischemic attack (0.80)  Asymptomatic cerebral infarction (5.40) | NA | 18.10 |
|  | clopidogrel 75mg QD+aspirin 81–100mg QD | Hypertension (81.70)  Dyslipidemia (82.00)  Diabetes (35.50)  Prior ischemic stroke (2.40)  Prior transient ischemic attack (0.50)  Asymptomatic cerebral infarction (7.80) | NA | 16.90 |
| Ohkubo K 2014 | clopidogrel 50mg QD +aspirin 100mg QD | Hypertension (71.00)  Hypercholesterolemia (88.00)  DM (46.00)  Previous MI (21.00) | Statins (84.00)  PPI (18.00) | 27.00 |
|  | clopidogrel 75mg QD +aspirin 100mg QD | Hypertension (68.0)  Hypercholesterolemia (90.00)  DM (41.00)  Previous MI (26.00) | Statins (83.00)  PPI (28.00) | 32.00 |
| Saito S 2014 | prasugrel 3.75mg QD +aspirin 81–100mg QD | Hypertension (72.30)  Dyslipidemia (75.30)  DM (36.50)  Prior MI (5.00) | PPI (41.20)  Statin (50.50)  Ca-blocker (26.90)  β-blocker (17.80) | 39.90 |
|  | clopidogrel 75mg QD +aspirin 81–100mg QD | Hypertension (72.40)  Dyslipidemia (73.70)  DM (35.00)  Prior MI (5.20) | PPI (42.30)  Statin (48.40)  Ca-blocker (23.50)  β-blocker (16.80) | 41.20 |
| Hiasa Y 2014 | ticagrelor 45mg BID+ aspirin 75–100mg QD | Hypertension (58.00)  Hyperlipidemia (52.00)  DM (16.00)  Prior MI (72.00) | NA | NA |
|  | ticagrelor 90mg BID+aspirin 75–100mg QD | Hypertension (67.00)  Hyperlipidemia (51.00)  DM (33.00)  Prior MI (84.00) | NA | NA |
|  | clopidogrel 75 mg QD+aspirin 75–100 mg QD | Hypertension (65.00)  Hyperlipidemia (50.00)  DM (26.00)  Prior MI (65.00) | NA | NA |
| Kitano D 2020 | prasugrel 3.75mg QD+aspirin 100mg QD | Hypertension (82.10)  DM (43.60)  Dyslipidemia (82.10) | NA | 25.60 |
|  | clopidogrel 75mg QD+aspirin 100mg QD | Hypertension (59.00)  DM (30.80)  Dyslipidemia (84.60) | NA | 48.70 |
| Wiviott SD 2005 | prasugrel 7.5mg QD+aspirin 325mg QD | DM (14.57)  Prior aspirin (40.20) | NA | 10.55 |
|  | prasugrel 10mg QD+aspirin 325mg QD | DM (12.50)  Prior aspirin (36.50) | NA | 12.50 |
| Wang H 2022 | ticagrelor 45 mg BID+ aspirin 100mg QD | Hypertension (61.30)  DM (43.50)  Dyslipidemia (48.40) | Statin (100.00) | 22.60 |
|  | ticagrelor 90 mg BID+ aspirin 100mg QD | Hypertension (63.80)  DM (48.30)  Dyslipidemia (53.40) | Statin (100.00) | 25.90 |
| Lu ZP 2022 | ticagrelor 60 mg BID+ aspirin 100mg QD | Hypertension (45.65)  DM (19.56) | NA | 45.65 |
|  | ticagrelor 90 mg BID+ aspirin 100mg QD | Hypertension (54.35)  DM (21.74) | NA | 47.83 |
| Wang JW 2021 | ticagrelor 60 mg BID+ aspirin 100mg QD | Hypertension (57.14)  DM (52.38)  Dyslipidemia (42.86) | NA | NA |
|  | clopidogrel 75mg QD+aspirin 100mg QD | Hypertension (59.52)  DM (47.62)  Dyslipidemia (38.10) | NA | NA |
| Ye JN 2021 | ticagrelor 60 mg BID+ aspirin 100mg QD | Hypertension (48.33)  DM (40.00)  Dyslipidemia (43.33) | β-blocker (65.00)  ACEI (78.33)  Statin (78.33)  Calcium channel blocker (18.33)  uragogue (10.00) | NA |
|  | ticagrelor 90 mg BID+ aspirin 100mg QD | Hypertension (53.33)  DM (41.67)  Dyslipidemia (40.00) | β-blocker (68.33)  ACEI (75.00)  Statin (81.67)  Calcium channel blocker (16.67)  uragogue (8.33) | NA |

CKD, chronic kidney disease; PVD, peripheral vessel disease; DM, diabetes mellitus; QD, quaque die; BID, bis in die; PPI, proton pump inhibitor; ACEI, angiotensin-converting enzyme inhibitor; ARB, angiotensin II receptor blocker; NA, not applicable.

# Supplementary Material 6: Risk of bias assessment

## 6.1 The results of risk of bias assessment for each study

**Table S6.1 The results of risk of bias assessment for each study**

| **Study** | **Randomization**  **process** | **Deviations from**  **intended**  **interventions** | **Missing outcome**  **data** | **Measurement of**  **the outcome** | **Selection of the**  **reported result** | **Overall** |
| --- | --- | --- | --- | --- | --- | --- |
| Jeong YH 2021 | 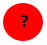 | 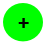 | 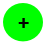 | 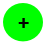 | 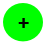 | 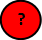 |
| Kim HS 2020 | 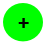 | 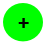 | 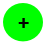 | 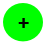 | 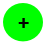 | 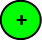 |
| Wang Y 2021 | 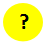 | 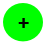 | 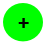 | 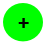 | 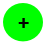 | 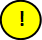 |
| Carrabba N 2016 | 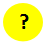 | 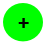 | 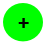 | 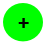 | 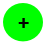 | 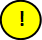 |
| Ueno T 2017 | 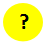 | 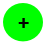 | 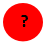 | 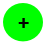 | 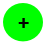 | 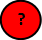 |
| Isshiki T 2014 | 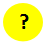 | 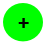 | 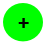 | 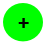 | 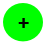 | 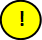 |
| Ohkubo K 2014 | 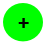 | 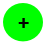 | 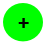 | 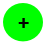 | 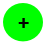 | 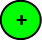 |
| Saito S 2014 | 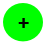 | 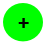 | 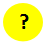 | 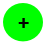 | 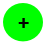 | 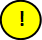 |
| Jin C 2017 | 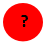 | 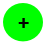 | 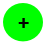 | 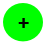 | 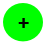 | 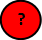 |
| Hiasa Y 2014 | 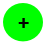 | 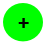 | 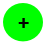 | 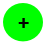 | 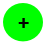 | 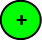 |
| Kitano D 2020 | 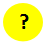 | 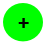 | 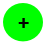 | 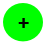 | 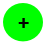 | 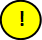 |
| Wiviott SD 2005 | 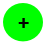 | 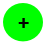 | 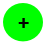 | 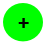 | 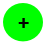 | 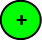 |
| Wang H 2022 | 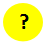 | 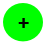 | 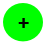 | 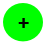 | 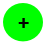 | 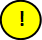 |
| Lu ZP 2022 | 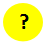 | 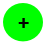 | 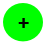 | 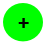 | 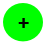 | 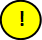 |
| Wang JW 2021 | 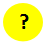 | 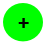 | 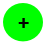 | 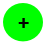 | 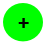 | 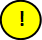 |
| Ye JN 2021 | 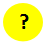 | 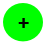 | 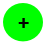 | 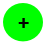 | 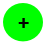 | 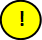 |


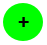
：Low risk;
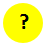
：Some concerns;
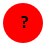
：High risk

**Figure S6.1 Risk of bias assessment of included studies**

## 6.2 The results of risk of bias assessment for each outcome

### 6.2.1 MACE_RoB_chart

**Figure S6.2.1 MACE_RoB_chart**

6.2.2 MI_RoB_chart

**Figure S6.2.2** MI_RoB_chart

6.2.3 Ischemic stroke_RoB_chart

**Figure S6.2.3** Ischemic stroke_RoB_chart

6.2.4 CVD_RoB_chart

**Figure S6.2.4** CVD_RoB_chart

6.2.5 ACD_RoB_chart

**Figure S6.2.5** ACD_RoB_chart

6.2.6 Bleeding_RoB_chart

**Figure S6.2.6** Bleeding_RoB_chart

6.2.7 Major bleeding_RoB_chart

**Figure S6.2.7** Major bleeding_RoB_chart

6.2.8 Minor bleeding_RoB_chart

**Figure S6.2.**8 Minor bleeding_RoB_chart

6.2.9 Minimal bleeding_RoB_chart

**Figure S6.2.**9 Minimal bleeding_RoB_chart

6.2.10 Bleeding events leading to discontinuation or non-adherence_RoB_chart

**Figure S**6.2.10 Bleeding events leading to discontinuation or non-adherence_RoB_chart

Supplementary Material 7: Trace plot and density plot, and Brooks-Gelman-Rubin diagnosis plot

## 7.1 The trace and density plot of MACE

**Figure S7.1. The trace and density plot of MACE**

## 7.2 The Brooks-Gelman-Rubin diagnosis plot of MACE

**Figure S7.2 The Brooks-Gelman-Rubin diagnosis plot of MACE**

## 7.3 The trace and density plot of MI

**Figure S7.3 The trace and density plot of MI**

## 7.4 The Brooks-Gelman-Rubin diagnosis plot of MI

**Figure S7.4 The Brooks-Gelman-Rubin diagnosis plot of M**I

## 7.5 The trace and density plot of ischaemic stroke

**Figure S7.5 The trace and density plot of ischaemic stroke**

## 7.6 The Brooks-Gelman-Rubin diagnosis plot of ischaemic stroke

**Figure S7.6 The Brooks-Gelman-Rubin diagnosis plot of ischaemic stroke**

## 7.7 The trace and density plot of CVD

**Figure S7.7 The trace and density plot of CVD**

## 7.8 The Brooks-Gelman-Rubin diagnosis plot of CVD

**Figure S7.8 The Brooks-Gelman-Rubin diagnosis plot of CVD**

## 7.9 The trace and density plot of ACD

**Figure S7.9 The trace and density plot of ACD**

## 7.10 The Brooks-Gelman-Rubin diagnosis plot of ACD

**Figure S7.10 The Brooks-Gelman-Rubin diagnosis plot of ACD**

## 7.11 The trace and density plot of bleeding

**Figure S7.11 The trace and density plot of bleeding**

## 7.12 The Brooks-Gelman-Rubin diagnosis plot of bleeding

**Figure S7.12 The Brooks-Gelman-Rubin diagnosis plot of bleeding**

## 7.13 The trace and density plot of major bleeding

**Figure S7.13** **The trace and density plot of major bleeding**

## 7.14 The Brooks-Gelman-Rubin diagnosis plot of major bleeding

**Figure S7.14 The Brooks-Gelman-Rubin diagnosis plot of major bleeding**

## 7.15 The trace and density plot of minor bleeding

**Figure S7.15 The trace and density plot of minor bleeding**

## 7.16 The Brooks-Gelman-Rubin diagnosis plot of minor bleeding

**Figure S7.16 The Brooks-Gelman-Rubin diagnosis plot of minor bleeding**

## 7.17 The trace and density plot of minimal bleeding

**Figure S7.17 The trace and density plot of minimal bleeding**

## 7.18 The Brooks-Gelman-Rubin diagnosis plot of minimal bleeding

**Figure S7.18 The Brooks-Gelman-Rubin diagnosis plot of minimal bleeding**

## 7.19 The trace and density plot of bleeding events leading to discontinuation or non-adherence

**Figure S7.19 The trace and density plot of bleeding events leading to discontinuation or non-adherence**

## 7.20 The Brooks-Gelman-Rubin diagnosis plot of bleeding events leading to discontinuation or non-adherence

**Figure S7.20 The Brooks-Gelman-Rubin diagnosis plot of bleeding discontinuatio**

# Supplementary Material 8: CINeMA Assessment

## 8.1 Confidence in effect estimates for MACE

**Table S8.1 Confidence in effect estimates for MACE**

| **Comparison** | **Number of studies** | **Within-study bias** | **Reporting bias** | **Indirectness** | **Imprecision** | **Heterogeneity** | **Incoherence** | **Confidence rating** |
| --- | --- | --- | --- | --- | --- | --- | --- | --- |
| Clopidogrel_standard:Prasugrel_low | 3 | Some concerns | Low risk | No concerns | Major concerns | No concerns | No concerns | Low |
| Clopidogrel_standard:Ticagrelor_low | 2 | Some concerns | Low risk | No concerns | Major concerns | No concerns | No concerns | Low |
| Prasugrel_low:Prasugrel_standard | 3 | Some concerns | Low risk | No concerns | Major concerns | No concerns | No concerns | Low |
| Prasugrel_low:Ticagrelor_low | 1 | Some concerns | Low risk | No concerns | Major concerns | No concerns | No concerns | Low |
| Ticagrelor_low:Ticagrelor_standard | 1 | Some concerns | Low risk | No concerns | Major concerns | No concerns | No concerns | Low |
| Clopidogrel_standard:Prasugrel_standard | 0 | Some concerns | Low risk | No concerns | Major concerns | No concerns | No concerns | Low |
| Clopidogrel_standard:Ticagrelor_standard | 0 | Some concerns | Low risk | No concerns | Major concerns | No concerns | No concerns | Low |
| Prasugrel_low:Ticagrelor_standard | 0 | Some concerns | Low risk | No concerns | Major concerns | No concerns | No concerns | Low |
| Prasugrel_standard:Ticagrelor_low | 0 | Some concerns | Low risk | No concerns | Major concerns | No concerns | No concerns | Low |
| Prasugrel_standard:Ticagrelor_standard | 0 | Some concerns | Low risk | No concerns | Major concerns | No concerns | No concerns | Low |

## 8.2 Confidence in effect estimates for MI

**Table S8.2 Confidence in effect estimates for MI**

| **Comparison** | **Number of studies** | **Within-study bias** | **Reporting bias** | **Indirectness** | **Imprecision** | **Heterogeneity** | **Incoherence** | **Confidence rating** |
| --- | --- | --- | --- | --- | --- | --- | --- | --- |
| Clopidogrel_standard:Prasugrel_low | 3 | Some concerns | Low risk | No concerns | Major concerns | No concerns | No concerns | Low |
| Clopidogrel_standard:Ticagrelor_low | 2 | Some concerns | Low risk | No concerns | Major concerns | No concerns | No concerns | Low |
| Prasugrel_low:Prasugrel_standard | 4 | No concerns | Low risk | No concerns | Major concerns | No concerns | No concerns | Low |
| Prasugrel_low:Ticagrelor_low | 1 | Some concerns | Low risk | No concerns | Major concerns | No concerns | No concerns | Low |
| Clopidogrel_standard:Prasugrel_standard | 0 | Some concerns | Low risk | No concerns | Major concerns | No concerns | No concerns | Low |
| Prasugrel_standard:Ticagrelor_low | 0 | Some concerns | Low risk | No concerns | Major concerns | No concerns | No concerns | Low |

## 8.3 Confidence in effect estimates for ischaemic stroke

**Table S8.3 Confidence in effect estimates for ischaemic stroke**

| **Comparison** | **Number of studies** | **Within-study bias** | **Reporting bias** | **Indirectness** | **Imprecision** | **Heterogeneity** | **Incoherence** | **Confidence rating** |
| --- | --- | --- | --- | --- | --- | --- | --- | --- |
| Clopidogrel_standard:Prasugrel_low | 4 | Some concerns | Low risk | No concerns | Major concerns | No concerns | Major concerns | Low |
| Prasugrel_low:Prasugrel_standard | 3 | No concerns | Low risk | No concerns | Major concerns | No concerns | Major concerns | Low |
| Clopidogrel_standard:Prasugrel_standard | 0 | Some concerns | Low risk | No concerns | Major concerns | No concerns | Major concerns | Low |

## 8.4 Confidence in effect estimates for CVD

**Table S8.4 Confidence in effect estimates for CVD**

| **Comparison** | **Number of studies** | **Within-study bias** | **Reporting bias** | **Indirectness** | **Imprecision** | **Heterogeneity** | **Incoherence** | **Confidence rating** |
| --- | --- | --- | --- | --- | --- | --- | --- | --- |
| Clopidogrel_standard:Prasugrel_low | 3 | Some concerns | Low risk | No concerns | Major concerns | No concerns | Major concerns | Low |
| Prasugrel_low:Prasugrel_standard | 3 | No concerns | Low risk | No concerns | Major concerns | No concerns | Major concerns | Low |
| Clopidogrel_standard:Prasugrel_standard | 0 | Some concerns | Low risk | No concerns | Major concerns | No concerns | Major concerns | Low |

## 8.5 Confidence in effect estimates for ACD

**Table S8.5 Confidence in effect estimates for ACD**

| **Comparison** | **Number of studies** | **Within-study bias** | **Reporting bias** | **Indirectness** | **Imprecision** | **Heterogeneity** | **Incoherence** | **Confidence rating** |
| --- | --- | --- | --- | --- | --- | --- | --- | --- |
| Clopidogrel_standard:Prasugrel_low | 3 | Some concerns | Low risk | No concerns | Major concerns | No concerns | Major concerns | Low |
| Prasugrel_low:Prasugrel_standard | 2 | No concerns | Low risk | No concerns | Major concerns | No concerns | Major concerns | Low |
| Clopidogrel_standard:Prasugrel_standard | 0 | Some concerns | Low risk | No concerns | Major concerns | No concerns | Major concerns | Low |

## 8.6 Confidence in effect estimates for bleeding

**Table S8.6 Confidence in effect estimates for bleeding**

| **Comparison** | **Number of studies** | **Within-study bias** | **Reporting bias** | **Indirectness** | **Imprecision** | **Heterogeneity** | **Incoherence** | **Confidence rating** |
| --- | --- | --- | --- | --- | --- | --- | --- | --- |
| Clopidogrel_low:Clopidogrel_standard | 1 | No concerns | Low risk | No concerns | Major concerns | No concerns | No concerns | Low |
| Clopidogrel_standard:Prasugrel_low | 3 | Some concerns | Low risk | No concerns | No concerns | Major concerns | No concerns | Low |
| Clopidogrel_standard:Ticagrelor_low | 3 | Some concerns | Low risk | No concerns | Major concerns | No concerns | No concerns | Low |
| Clopidogrel_standard:Ticagrelor_standard | 1 | Some concerns | Low risk | No concerns | No concerns | No concerns | No concerns | Moderate |
| Prasugrel_low:Prasugrel_standard | 1 | Some concerns | Low risk | No concerns | No concerns | Major concerns | No concerns | Low |
| Prasugrel_low:Ticagrelor_low | 1 | Some concerns | Low risk | No concerns | Major concerns | No concerns | No concerns | Low |
| Ticagrelor_low:Ticagrelor_standard | 3 | Some concerns | Low risk | No concerns | No concerns | Major concerns | No concerns | Low |
| Clopidogrel_low:Prasugrel_low | 0 | Some concerns | Low risk | No concerns | Major concerns | No concerns | No concerns | Low |
| Clopidogrel_low:Prasugrel_standard | 0 | Some concerns | Low risk | No concerns | Major concerns | No concerns | No concerns | Low |
| Clopidogrel_low:Ticagrelor_low | 0 | Some concerns | Low risk | No concerns | Major concerns | No concerns | No concerns | Low |
| Clopidogrel_low:Ticagrelor_standard | 0 | No concerns | Low risk | No concerns | Major concerns | No concerns | No concerns | Low |
| Clopidogrel_standard:Prasugrel_standard | 0 | Some concerns | Low risk | No concerns | No concerns | No concerns | No concerns | Moderate |
| Prasugrel_low:Ticagrelor_standard | 0 | Some concerns | Low risk | No concerns | Major concerns | No concerns | No concerns | Low |
| Prasugrel_standard:Ticagrelor_low | 0 | Some concerns | Low risk | No concerns | Major concerns | No concerns | No concerns | Low |
| Prasugrel_standard:Ticagrelor_standard | 0 | Some concerns | Low risk | No concerns | Major concerns | No concerns | No concerns | Low |

## 8.7 Confidence in effect estimates for major bleeding

**Table S8.7 Confidence in effect estimates for major bleeding**

| **Comparison** | **Number of studies** | **Within-study bias** | **Reporting bias** | **Indirectness** | **Imprecision** | **Heterogeneity** | **Incoherence** | **Confidence rating** |
| --- | --- | --- | --- | --- | --- | --- | --- | --- |
| Clopidogrel_standard:Prasugrel_low | 3 | Some concerns | Low risk | No concerns | Major concerns | No concerns | Major concerns | Low |
| Prasugrel_low:Prasugrel_standard | 3 | No concerns | Low risk | No concerns | Major concerns | No concerns | Major concerns | Low |
| Clopidogrel_standard:Prasugrel_standard | 0 | Some concerns | Low risk | No concerns | Major concerns | No concerns | Major concerns | Low |

## 8.8 Confidence in effect estimates for minor bleeding

**Table S8.8 Confidence in effect estimates for minor bleeding**

| **Comparison** | **Number of studies** | **Within-study bias** | **Reporting bias** | **Indirectness** | **Imprecision** | **Heterogeneity** | **Incoherence** | **Confidence rating** |
| --- | --- | --- | --- | --- | --- | --- | --- | --- |
| Clopidogrel_standard:Prasugrel_low | 1 | Some concerns | Low risk | No concerns | Major concerns | No concerns | No concerns | Low |
| Clopidogrel_standard:Ticagrelor_low | 2 | Some concerns | Low risk | No concerns | Major concerns | No concerns | No concerns | Low |
| Clopidogrel_standard:Ticagrelor_standard | 1 | Some concerns | Low risk | No concerns | Major concerns | No concerns | No concerns | Low |
| Prasugrel_low:Prasugrel_standard | 2 | Some concerns | Low risk | No concerns | Major concerns | No concerns | No concerns | Low |
| Ticagrelor_low:Ticagrelor_standard | 5 | Some concerns | Low risk | No concerns | No concerns | No concerns | No concerns | Moderate |
| Clopidogrel_standard:Prasugrel_standard | 0 | Some concerns | Low risk | No concerns | Major concerns | No concerns | No concerns | Low |
| Prasugrel_low:Ticagrelor_low | 0 | Some concerns | Low risk | No concerns | Major concerns | No concerns | No concerns | Low |
| Prasugrel_low:Ticagrelor_standard | 0 | Some concerns | Low risk | No concerns | Major concerns | No concerns | No concerns | Low |
| Prasugrel_standard:Ticagrelor_low | 0 | Some concerns | Low risk | No concerns | Major concerns | No concerns | No concerns | Low |
| Prasugrel_standard:Ticagrelor_standard | 0 | Some concerns | Low risk | No concerns | Major concerns | No concerns | No concerns | Low |

## 8.9 Confidence in effect estimates for minimal bleeding

**Table S8.** **9 Confidence in effect estimates for minimal bleeding**

| **Comparison** | **Number of studies** | **Within-study bias** | **Reporting bias** | **Indirectness** | **Imprecision** | **Heterogeneity** | **Incoherence** | **Confidence rating** |
| --- | --- | --- | --- | --- | --- | --- | --- | --- |
| Clopidogrel_standard:Prasugrel_low | 1 | Some concerns | Low risk | No concerns | Major concerns | No concerns | No concerns | Low |
| Clopidogrel_standard:Ticagrelor_low | 3 | Some concerns | Low risk | No concerns | Major concerns | No concerns | No concerns | Low |
| Clopidogrel_standard:Ticagrelor_standard | 1 | No concerns | Low risk | No concerns | No concerns | Major concerns | No concerns | Low |
| Prasugrel_low:Prasugrel_standard | 3 | Some concerns | Low risk | No concerns | No concerns | No concerns | No concerns | Moderate |
| Prasugrel_low:Ticagrelor_low | 1 | Major concerns | Low risk | No concerns | Major concerns | No concerns | No concerns | Very Low |
| Ticagrelor_low:Ticagrelor_standard | 2 | No concerns | Low risk | No concerns | Major concerns | No concerns | No concerns | Low |
| Clopidogrel_standard:Prasugrel_standard | 0 | Some concerns | Low risk | No concerns | Major concerns | No concerns | No concerns | Low |
| Prasugrel_low:Ticagrelor_standard | 0 | Some concerns | Low risk | No concerns | Major concerns | No concerns | No concerns | Low |
| Prasugrel_standard:Ticagrelor_low | 0 | Some concerns | Low risk | No concerns | Major concerns | No concerns | No concerns | Low |
| Prasugrel_standard:Ticagrelor_standard | 0 | Some concerns | Low risk | No concerns | Major concerns | No concerns | No concerns | Low |

## 8.10 Confidence in effect estimates for bleeding events leading to discontinuation or non-adherence

**Table S8.10 Confidence in effect estimates for bleeding events leading to discontinuation or non-adherence**

| **Comparison** | **Number of studies** | **Within-study bias** | **Reporting bias** | **Indirectness** | **Imprecision** | **Heterogeneity** | **Incoherence** | **Confidence rating** |
| --- | --- | --- | --- | --- | --- | --- | --- | --- |
| Clopidogrel_standard:Prasugrel_low | 2 | Some concerns | Low risk | No concerns | Major concerns | No concerns | Major concerns | Low |
| Prasugrel_low:Prasugrel_standard | 1 | No concerns | Low risk | No concerns | No concerns | Major concerns | Major concerns | Low |
| Clopidogrel_standard:Prasugrel_standard | 0 | Some concerns | Low risk | No concerns | No concerns | Major concerns | Major concerns | Low |

# Supplementary Material 9: Network plot

## 9.1 Ischaemic stroke

**Figure S9.1 Network plot of ischaemic stroke**

## 9.2 CVD

**Figure S9.2 Network plot of CVD**

## 9.3 ACD

**Figure S9.3 Network plot of ACD**

## 9.4 Minor bleeding

**Figure S9.4 Network plot of minor bleeding**

## 9.5 Minimal bleeding

**Figure S9.5 Network plot of minimal bleeding**

## 9.6 Bleeding events leading to discontinuation or non-adherence

**Figure S9.6 Network plot of bleeding events leading to discontinuation or non-adherence**

# Supplementary Material 10: league table for network meta-analysis

## 10.1 Network meta-analysis results for MI

| Prasugrel_low | 1.77 (0.04, 79.39) | 1.48 (0.71, 3.89) | 1.00 (0.39, 2.50) |
| --- | --- | --- | --- |
| 0.56 (0.01, 22.34) | Ticagrelor_low | 0.85 (0.02, 32.42) | 0.56 (0.01, 24.09) |
| 0.68 (0.26, 1.42) | 1.18 (0.03, 52.03) | Clopidogrel_standard | 0.67 (0.17, 2.12) |
| 1.00 (0.4, 2.56) | 1.78 (0.04, 94.43) | 1.49 (0.47, 5.72) | Prasugrel_standard |

Treatment estimates are ORs and 95% CIs. Signifificant results are italicized and highlighted in bold；OR = odds ratio.

##

## 10.2 Network meta-analysis results for ischaemic stroke

| Prasugrel_standard | 1.01 (0.16, 5.08) | 0.98 (0.10, 7.14) |
| --- | --- | --- |
| 0.99 (0.20, 6.10) | Prasugrel_low | 0.98 (0.26, 3.22) |
| 1.02 (0.14, 9.96) | 1.02 (0.31, 3.88) | Clopidogrel_standard |

Treatment estimates are ORs and 95% CIs. Signifificant results are italicized and highlighted in bold；OR = odds ratio.

## 10.3 Network meta-analysis results for CVD

| Prasugrel_standard | 0.26 (0.04, 1.53) | 0.20 (0.02, 2.12) |
| --- | --- | --- |
| 3.78 (0.66, 27.23) | Prasugrel_low | 0.75 (0.15, 3.74) |
| 5.05 (0.47, 66.52) | 1.33 (0.27, 6.71) | Clopidogrel_standard |

Treatment estimates are ORs and 95% CIs. Signifificant results are italicized and highlighted in bold；OR = odds ratio.

## 10.4 Network meta-analysis results for ACD

| Prasugrel_standard | 0.60(0.12, 2.18) | 0.51 (0.06, 3.01) |
| --- | --- | --- |
| 1.68 (0.46, 8.27) | Prasugrel_low | 0.86 (0.21, 2.99) |
| 1.97 (0.33, 17.59) | 1.17 (0.33, 4.70) | Clopidogrel_standard |

Treatment estimates are ORs and 95% CIs. Signifificant results are italicized and highlighted in bold；OR = odds ratio.

## 10.5 Network meta-analysis results for major bleeding

| Prasugrel_standard | 1.29 (0.05, 34.54) | 8.94 (0.18, 1442.30) |
| --- | --- | --- |
| 0.77 (0.03, 19.74) | Prasugrel_low | 6.87 (0.60, 283.95) |
| 0.11 (0.00, 5.43) | 0.15 (0.00, 1.66) | Clopidogrel_standard |

Treatment estimates are ORs and 95% CIs. Signifificant results are italicized and highlighted in bold；OR = odds ratio.

## 10.6 Network meta-analysis results for minor bleeding

| Prasugrel_low | 1.23 (0.30, 5.07) | 1.29 (0.07, 51.32) | 3.65 (0.18, 153.30) | 0.55 (0.17, 1.72) |
| --- | --- | --- | --- | --- |
| 0.81 (0.20, 3.33) | Prasugrel_standard | 1.09 (0.04, 51.25) | 3.08 (0.10, 154.31) | 0.44 (0.07, 2.74) |
| 0.77 (0.02, 13.84) | 0.92 (0.02, 23.38) | Ticagrelor_low | 2.78 (1.22, 6.50) | 0.42 (0.01, 6.28) |
| 0.27 (0.01, 5.68) | 0.32 (0.01, 9.63) | 0.36 (0.15, 0.82) | Ticagrelor_standard | 0.15 (0.00, 2.48) |
| 1.83 (0.58, 5.97) | 2.29 (0.36, 14.03) | 2.36 (0.16, 82.07) | 6.73 (0.40, 260.83) | Clopidogrel_standard |

Treatment estimates are ORs and 95% CIs. Signifificant results are italicized and highlighted in bold；OR = odds ratio.

## 10.7 Network meta-analysis results for minimal bleeding

| Prasugrel_standard | 0.41 (0.17, 0.87) | 0.57 (0.03, 24.81) | 1.09 (0.05, 53.48) | 0.40 (0.02, 18.07) |
| --- | --- | --- | --- | --- |
| 2.41 (1.15, 5.78) | Prasugrel_low | 1.36 (0.09, 54.25) | 2.61 (0.15, 123.64) | 0.97 (0.06, 39.30) |
| 1.77 (0.04, 30.08) | 0.73 (0.02, 10.63) | Ticagrelor_low | 1.91 (0.66, 5.63) | 0.72 (0.24, 1.76) |
| 0.92 (0.02, 19.56) | 0.38 (0.01, 6.71) | 0.52 (0.18, 1.51) | Ticagrelor_standard | 0.37 (0.10, 1.17) |
| 2.51 (0.06, 46.53) | 1.03 (0.03, 16.76) | 1.40 (0.57, 4.16) | 2.68 (0.86, 10.26) | Clopidogrel_standard |

Treatment estimates are ORs and 95% CIs. Signifificant results are italicized and highlighted in bold；OR = odds ratio.

## 10.8 Network meta-analysis results for bleeding events leading to discontinuation or non-adherence

| Prasugrel_low | 3.38 (0.86, 14.27) | 1.22 (0.43, 3.37) |
| --- | --- | --- |
| 0.30 (0.07, 1.17) | Prasugrel_standard | 0.36 (0.06, 2.04) |
| 0.82 (0.30, 2.31) | 2.78 (0.49, 16.19) | Clopidogrel_standard |

Treatment estimates are ORs and 95% CIs. Signifificant results are italicized and highlighted in bold；OR = odds ratio.

**Supplementary Material 11: Forest plot of network meta-analysis**

## 11.1 Ischaemic stroke

**Figure S11.1 Forest plot of ischaemic stroke**

## 11.2 CVD

**Figure S11.2 Forest plot of CVD**

## 11.3 ACD

**Figure S11.3 Forest plot of ACD**

## 11.4 Minor bleeding

**Figure S11.4 Forest plot of minor bleeding**

## 11.5 Minimal bleeding

**Figure S11.5 Forest plot of minimal bleeding**

## 11.6 Bleeding events leading to discontinuation or non-adherence

**Figure S11.6 Forest plot of bleeding events leading to discontinuation or non-adherence**

# Supplementary Material 12: Contribution plots by study outcome

## 12.1 Contribution plot for MACE

**Figure S12.1 Contribution plot for MACE**

## 12.2 Contribution plot for MI

**Figure S12.2 Contribution plot for MI**

## 12.3 Contribution plot for ischaemic stroke

**Figure S12.3 Contribution plot for ischaemic stroke**

## 12.4 Contribution plot for CVD

**Figure S12.4 Contribution plot for CVD**

## 12.5 Contribution plot for ACD

**Figure S12.5 Contribution plot for ACD**

## 12.6 Contribution plot for bleeding

**Figure S12.6 Contribution plot for bleeding**

## 12.7 Contribution plot for major bleeding

**Figure S12.7 Contribution plot for major bleeding**

## 12.8 Contribution plot for minor bleeding

**Figure S12.8 Contribution plot for minor bleeding**

## 12.9 Contribution plot for minimal bleeding

**Figure S12.9 Contribution plot for minimal bleeding**

## 12.10 Contribution plot for bleeding events leading to discontinuation or non-adherence

**Figure S12.10 Contribution plot for bleeding events leading to discontinuation or non-adherence**

Supplementary Material 13: Treatment Ranking using SUCRA

## 13.1 Cumulative ranking curves for ischaemic stroke

**Figure S13.1Cumulative ranking curves for ischaemic stroke**

## 13.2 Cumulative ranking curves for CVD

**Figure S13.2 Cumulative ranking curves for CVD**

## 13.3 Cumulative ranking curves for ACD

**Figure S13.3 Cumulative ranking curves for ACD**

## 13.4 Cumulative ranking curves for minor bleeding

**Figure S13.4 Cumulative ranking curves for minor bleeding**

## 13.5 Cumulative ranking curves for minimal bleeding

**Figure S13.5 Cumulative ranking curves for minimal bleeding**

## 13.6 Cumulative ranking curves for bleeding events leading to discontinuation or non-adherence

**Figure S13.6 Cumulative ranking curves for bleeding events leading to discontinuation or non-adherence**

#

# Supplementary Material 14: Subgroup in patients with ACS

## 14.1 Forest for MACE in patients with ACS

**Figure S14.1 Forest for MACE in patients with ACS**

## 14.2 Forest for MI in patients with ACS

**Figure S14.2 Forest for MI in patients with ACS**

## 14.3 Forest for ischaemic stroke in patients with ACS

**Figure S14.3 Forest for ischaemic stroke in patients with ACS**

## 14.4 Forest for CVD in patients with ACS

**Figure S14.4 Forest for CVD in patients with ACS**

## 14.5 Forest for ACD in patients with ACS

**Figure S14.5 Forest for ACD in patients with ACS**

## 14.6 Forest for bleeding in patients with ACS

**Figure S14.6 Forest for bleeding in patients with ACS**

## 14.7 Forest for major bleeding in patients with ACS

**Figure S14.7 Forest for major bleeding in patients with ACS**

## 14.8 Forest for minor bleeding in patients with ACS

**Figure S14.8 Forest for minor bleeding in patients with ACS**

## 14.9 Forest for minimal bleeding in patients with ACS

**Figure S14.9 Forest for minimal bleeding in patients with ACS**

# Supplementary Material 15: Subgroup in Asia

## 15.1 Forest for MACE in Asia

**Figure S15.1 Forest for MACE in Asia**

## 15.2 Forest for MI in Asia

**Figure S15.2 Forest for MI in Asia**

## 15.3 Forest for ischaemic stroke in Asia

**Figure S15.3 Forest for ischaemic stroke in Asia**

## 15.4 Forest for CVD in Asia

**Figure S15.4 Forest for CVD in Asia**

## 15.5 Forest for ACD in Asia

**Figure S15.5 Forest for ACD in Asia**

## 15.6 Forest for bleeding in Asia

**Figure S15.6 Forest for bleeding in Asia**

## 15.7 Forest for major bleeding in Asia

**Figure S15.7 Forest for major bleeding in Asia**

## 15.8 Forest for minor bleeding in Asia

**Figure S15.8 Forest for minor bleeding in Asia**

## 15.9 Forest for minimal bleeding in Asia

**Figure S15.9 Forest for minimal bleeding in Asia**

# Supplementary Material 16: Subgroup in patients with BMI of 24-26kg/m^2^

## 16.1 Forest for MACE in patients with BMI of 24-26kg/m^2^

**Figure S16.1 Forest for MACE in patients with BMI of 24-26kg/m^2^**

## 16.2 Forest for MI in patients with BMI of 24-26kg/m^2^

**Figure S16.2 Forest for MI in patients with BMI of 24-26kg/m^2^**

## 16.3 Forest for ischaemic stroke in patients with BMI of 24-26kg/m^2^

**Figure S16.3 Forest for ischaemic stroke in patients with BMI of 24-26kg/m^2^**

## 16.4 Forest for CVD in patients with BMI of 24-26kg/m^2^

**Figure S16.4 Forest for CVD in patients with BMI of 24-26kg/m^2^**

## 16.5 Forest for ACD in patients with BMI of 24-26kg/m^2^

**Figure S16.5 Forest for ACD in patients with BMI of 24-26kg/m^2^**

## 16.6 Forest for bleeding in patients with BMI of 24-26kg/m^2^

**Figure S16.6 Forest for bleeding in patients with BMI of 24-26kg/m^2^**

## 16.7 Forest for major bleeding in patients with BMI of 24-26kg/m^2^

**Figure S16.7 Forest for major bleeding in patients with BMI of 24-26kg/m^2^**

## 16.8 Forest for minor bleeding in patients with BMI of 24-26kg/m^2^

**Figure S16.8 Forest for minor bleeding in patients with BMI of 24-26kg/m^2^**

## 16.9 Forest for minimal bleeding in patients with BMI of 24-26kg/m^2^

**Figure S16.9 Forest for minimal bleeding in patients with BMI of 24-26kg/m^2^**

# Supplementary Material 17: Comparison-adjusted funnel plots

## 17.1 Comparison adjusted funnel plot for MACE

**Figure S17.1 Comparison adjusted funnel plot for MACE**

## 17.2 Comparison adjusted funnel plot for MI

**Figure S17.2 Comparison adjusted funnel plot for MI**

## 17.3 Comparison adjusted funnel plot for ischaemic stroke

**Figure S17.3 Comparison adjusted funnel plot for ischaemic stroke**

## 17.4 Comparison adjusted funnel plot for CVD

**Figure S17. 4 Comparison adjusted funnel plot for CVD**

## 17.5 Comparison adjusted funnel plot for ACD

**Figure S17. 5 Comparison adjusted funnel plot for ACD**

## 17.6 Comparison adjusted funnel plot for bleeding

**Figure S17.6 Comparison adjusted funnel plot for bleeding**

## 17.7 Comparison adjusted funnel plot for major bleeding

**Figure S17.7 Comparison adjusted funnel plot for major bleeding**

## 17.8 Comparison adjusted funnel plot for minor bleeding

##

**Figure S17.8 Comparison adjusted funnel plot for minor bleeding**

## 17.9 Comparison adjusted funnel plot for minimal bleeding

**Figure S17.9 Comparison adjusted funnel plot for minimal bleeding**

## 17.10 Comparison adjusted funnel plot for bleeding events leading to discontinuation or non-adherence

**Figure S17.10 Comparison adjusted funnel plot for bleeding events leading to discontinuation or non-adherence**
